# Assessment of the human response to acute mental stress–An overview and a multimodal study

**DOI:** 10.1371/journal.pone.0294069

**Published:** 2023-11-09

**Authors:** Hannes Ernst, Matthieu Scherpf, Sebastian Pannasch, Jens R. Helmert, Hagen Malberg, Martin Schmidt

**Affiliations:** 1 Institute of Biomedical Engineering, TU Dresden, Dresden, Germany; 2 Chair of Engineering Psychology and Applied Cognitive Research, TU Dresden, Dresden, Germany; VitalConnect (United States), UNITED STATES

## Abstract

Numerous vital signs are reported in association with stress response assessment, but their application varies widely. This work provides an overview over methods for stress induction and strain assessment, and presents a multimodal experimental study to identify the most important vital signs for effective assessment of the response to acute mental stress. We induced acute mental stress in 65 healthy participants with the Mannheim Multicomponent Stress Test and acquired self-assessment measures (Likert scale, Self-Assessment Manikin), salivary α-amylase and cortisol concentrations as well as 60 vital signs from biosignals, such as heart rate variability parameters, QT variability parameters, skin conductance level, and breath rate. By means of statistical testing and a self-optimizing logistic regression, we identified the most important biosignal vital signs. Fifteen biosignal vital signs related to ventricular repolarization variability, blood pressure, skin conductance, and respiration showed significant results. The logistic regression converged with QT variability index, left ventricular work index, earlobe pulse arrival time, skin conductance level, rise time and number of skin conductance responses, breath rate, and breath rate variability (F1 = 0.82). Self-assessment measures indicated successful stress induction. α-amylase and cortisol showed effect sizes of -0.78 and 0.55, respectively. In summary, the hypothalamic-pituitary-adrenocortical axis and sympathetic nervous system were successfully activated. Our findings facilitate a coherent and integrative understanding of the assessment of the stress response and help to align applications and future research concerning acute mental stress.

## 1. Introduction

Acute stress is an everyday phenomenon and the human response to it represents an essential survival mechanism [1]. It activates the organism and leads to the short-term provision of energy reserves [1]. Repeated or prolonged exposure, however, leads to severe negative effects on human health as chronic stress causes coronary heart disease [2, 3] and hypertension [3, 4] and is associated with depression [5], atherosclerosis [6], and other pathologies [1]. Additionally, acute stress acts as a trigger for cardiac events such as myocardial infarction or sudden cardiac death [2, 3]. Therefore, methods for the assessment of acute stress are of general interest and many different techniques are applied in clinical and laboratory contexts. However, to date, there is no uniform standard to quantify acute mental stress or strain [7]. Typical techniques for the assessment of the human stress response target behavioral patterns, the hypothalamic-pituitary-adrenocortical (HPA) axis, and the sympathoadrenal system [7–9]. Behavioral patterns can be registered with questionnaires while monitoring of the HPA axis and the sympathoadrenal system require biomedical engineering techniques to acquire vital signs and chemical biomarkers. Especially when it comes to vital signs derived from biosignals, researchers often rely on only a few measures, the selection of which considerably varies (see [10, 11] for example). This hampers an integrative understanding of stress responses. To address this issue, we cover two aspects in this work. First, we provide a fundamental overview over methods for stress induction and stress response assessment. Second, we present a multimodal experimental study for the assessment of the physiological stress response to identify the most important vital signs with regard to acute mental stress. The multimodal approach allows for the derivation of a wide range of vital signs within the same participant group, thus enabling an integrative discussion. The overview is outlined in chapter 3. In chapter 4, the study design is described. Chapter 5 presents the results of the study. In chapter 6, we discuss our findings in the context of the state of the art and chapter 7 provides a summary together with an outlook for future directions to foster a more unified assessment of the response to acute mental stress. Future research shall address the dataset of this study by the name “Dresden Multimodal Biosignal Dataset for the Mannheim Multicomponent Stress Test” (DMBD-MMST).

## 2. Overview and related work

Over time, numerous descriptions for stress evolved, which frequently blurs demarcation of research [12]. Therefore, this overview begins with a definition of mental stress as it is understood at present. Subsequently, we provide a collection of clinical and laboratory methods for stress induction. The physiology of the human response to acute mental stress in terms of the sympathoadrenal system and the HPA axis is described briefly followed by clinical and laboratory methods for the assessment of the stress response. Finally, biosignals suitable for stress response assessment are introduced and relevant vital signs are discussed in more detail.

### 2.1. Definition of stress and strain

Today, the International Organization for Standardization (ISO) defines *mental stress* in ISO 10075-1 [13] as the

“total of all assessable influences impinging upon a human being from external sources and affecting that person mentally.” [13 p. 2]

This definition comes from an ergonomic point of view and is congruent with the term *work stress* in ISO 6385 [14]. Impinging factors, often called stressors, take many forms including environmental conditions, societal and organizational factors, and task requirements [13].

A less common but much more direct term for what is typically targeted in stress assessment is *mental strain*, the

“immediate effect of mental stress within the individual depending on their current condition.”[13 p. 2]

**Fig 1:**
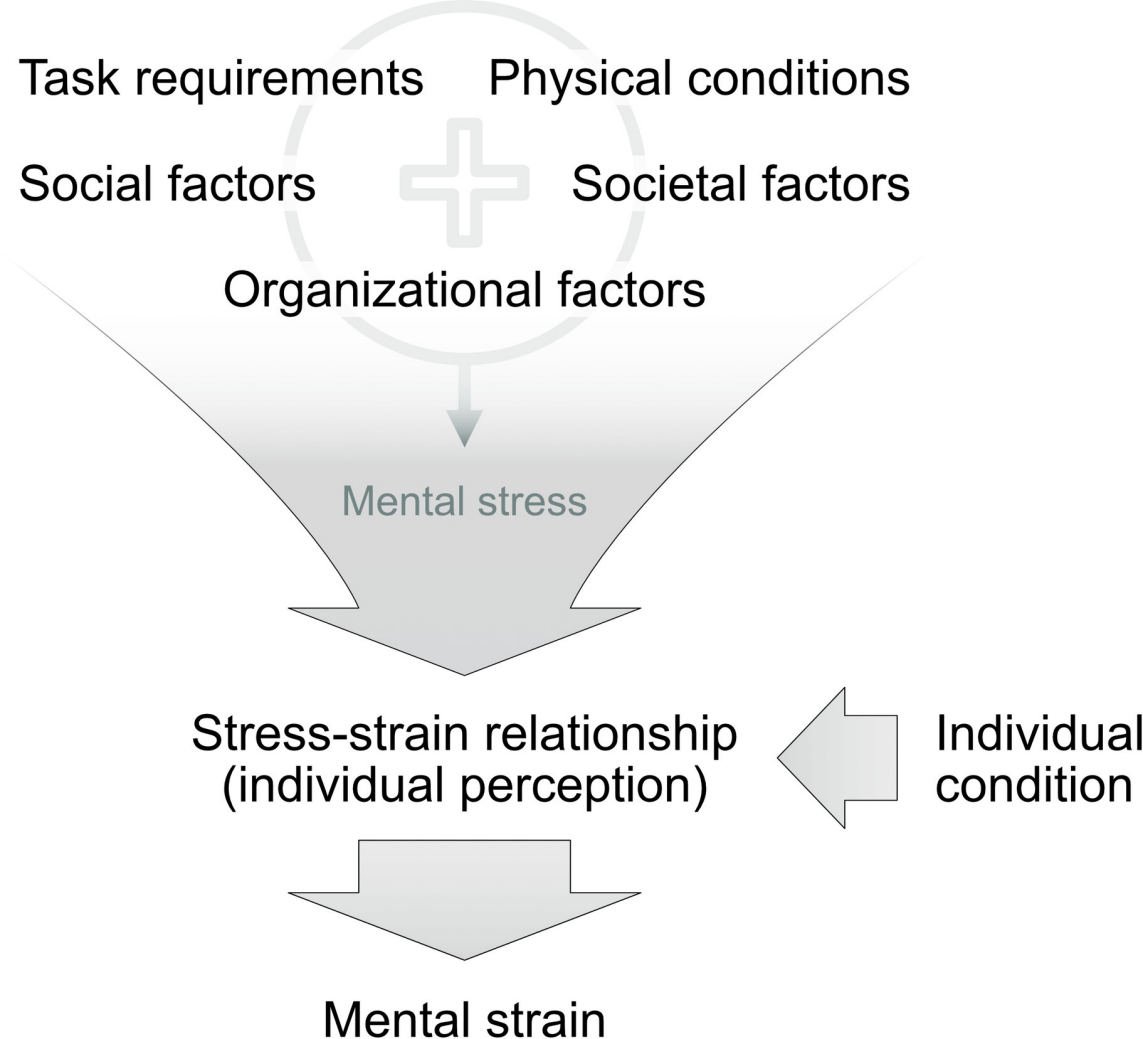
ISO 10075-1 terminology for mental workload.

This means that identical stressors may elicit different responses even in the same individual depending on their appraisal. For example, a task may be challenging at first but become less burdensome when the individual learns how to cope with it. Fig 1 illustrates the principle of mental stress and strain [13].

*Acute stress* refers to a transient condition, i.e. stressor intensity decreases after some time (up to a few hours), with repercussions that decay with appropriate recuperation, while *chronic stress* refers to repeated or prolonged periods (up to months or years) of high stressor intensity with repercussions persisting even after the stressor [13].

ISO 10075-1 utilizes the term *mental workload* as a generic descriptor for both mental stress and strain [13]. This exemplifies the conceptual vagueness of the literature, since mental workload can also be understood in the sense of cognitive load as strictly task-related limiting the impinging influences to the task requirements [15]. In this understanding, the stress-strain relationship mainly depends on the available processing capacity with respect to the task, which may be affected by the individuals’ condition (e.g. drowsiness). Other impinging influences come to be of secondary importance.

To clarify, this work employs the terminology of ISO 10075-1. In this sense, if a vital sign such as heart rate is measured to investigate the effect of a stressor, this is to be addressed as strain assessment or assessment of the stress response. To assess stress from the vital sign, all aspects of the individuals’ condition would have to be taken into account to model the stress-strain relationship [16], which is often unfeasible.

### 2.2. Methods for stress induction

Over time, numerous methods for controlled stressful stimulation have been developed. Table 1 provides an overview over well-known methods not limited to mental stress. In addition to the listed methods, there are variations and specialized methods for specific research objectives. For other comparative overviews, the reader is referred to [9, 11, 17, 18].

**Table 1:** Overview over clinical and laboratory methods to induce acute stress. Methods are sorted by stressor type and ordered alphabetically by name.

Source	Name	Summary	Stressor Type	Coping	Social Threat
[21]	Berg Card Sorting Test (BCST)	Sorting drawn cards into one of four open card piles depending on shape, number, and color of the pattern with feedback by study supervisor after each card. Sorting criterion unknown to participant and changed when found out.	mental	active	yes
[22]	Computer Games and Puzzle Solving Tasks	Playing challenging computer games like Tetris or Mortal Combat at high difficulty.	mental	active	no
[23]	Computerized Short Version of the Berg Card Sorting Test	Shortened version of the Berg Card Sorting Test that is performed at a computer.	mental	active	no
[24, 25]	Mannheim Multicomponent Stress Test (MMST)	Computerized Paced Auditory Serial Addition Task with affective background images, swelling white noise, negative acoustic feedback, motivational stressors and decreasing answer time.	mental	active	no
[26]	Mirror Tracing	Drawing a predestined pattern (e.g. a star). The drawing can only be viewed in a mirror.	mental	active	no
[27]	Montreal Imaging Stress Task (MIST)	Mental arithmetic task (basic operations) manipulated beyond mental capacity, information on performance as well as personal evaluation from study supervisors.	mental	active	yes
[28]	Movie Watching	Watching traumatizing video clips in a dark room.	mental	passive	yes
[29]	*n*-back Task	Memorizing the state *n* steps beforehand (e.g. a light that switches positions or a character in a sequence of characters).	mental	active	no
[30, 31]	Paced Auditory Serial Addition Task (PASAT)	Adding the last two numbers of a numerical sequence presented with increasing speed.	mental	active	no^*^
[32]	Psycho-Physiological-Stress-Test (PPST)	Identifying numbers in a large matrix with visible and acoustic performance feedback in several difficulties.	mental	active	no
[33]	Simple Singing Stress Procedure (SSSP)	Participants have to sing two songs to the study supervisor who records and evaluates the performance (song category assigned directly before performance). In between the two songs, a cognitive task may be placed to investigate the effect of strain on the task.	mental	active	yes
[34]	Sing-a-Song Stress Test (SSST)	Participants consecutively read nine neutral phrases on a monitor; the tenth phrase commands the task to sing a song after a counter has expired.	mental	passive	no^†^
[35]	Stroop Color and Word Test	Reading out words or reciting the print color of words. Words are color names with incongruence between a word and the color it is printed in.	mental	active	no^§^
[36]	Trier Social Stress Test (TSST)	Combination of free speech in front of an audience of study supervisors and a mental arithmetic task including negative feedback by the audience.	mental	active	yes
[37]	Wisconsin Card Sorting Test (WCST)	See Berg Card Sorting Test.	mental	active	yes
[38]	Maastricht Acute Stress Test (MAST)	Prolonged Socially Evaluated Cold Pressure Test but with a mental arithmetic task during the resting periods including negative feedback (similar to Trier Social Stress Test).	both	both	yes
[39]	6-minute Walk	6 min walk for people who cannot perform cycle ergometer or treadmill exercise.	physical	active	no
[40, 41]	CO_2_ Challenge	Inhalation of gas with different carbon dioxide concentrations.	physical	passive	no
[42]	Cold Pressure Test (CPT)	Holding a limb in ice water (4 – 5°C).	physical	passive	no
[39]	Cycle Ergometer Exercise	Cycling on an ergometer with increasing load.	physical	active	no
[43]	Head-Up Tilt Table Test	Passive change of the participants’ posture from supine to almost orthostatic.	physical	passive	no
[44]	Heat Tolerance Test	Exercise in hot environment (40°C).	physical	active	no
[38]	Prolonged Socially Evaluated Cold Pressure Test (PSECPT)	Socially Evaluated Cold Pressure Test but with multiple immersions and short resting periods. Timing dictated by a computer.	physical	passive	yes
[45]	Socially Evaluated Cold Pressure Test (SECPT)	Cold Pressure Test in which participants are videotaped and closely monitored by a study supervisor of opposite sex who lacks empathy.	physical	passive	yes
[39]	Treadmill Exercise	Walking on treadmill with adjusting speed, grade, stage duration and oxygen uptake.	physical	active	no

^*^ Originally, study supervisors in close proximity to participants, but errors triggered no response. Modern computerized versions do not require study supervisors near the participant.

^†^ Given that no other persons are near the participant.

^§^ Originally, study supervisors pointed out errors in person, which means there was a social-evaluative threat. However, modern computerized versions usually spare this interaction.

Clinical examinations mainly target physical stressors of bodily exertion (e.g. cycle ergometer exercise), while mental stressors dominate in psychophysiological research (e.g. Trier Social Stress Test). Some methods require active coping, which means participants must take action in some kind (e.g. solve a task, exert motion) [19]. Methods that require passive coping do not require participants to take action but to persevere and endure the stressful stimulation (e.g. noise, cold) [19].

A meta-analysis investigating cortisol responses to acute mental stress identified factors that elicit particularly strong stress responses [20]: social-evaluative threat, novelty, uncontrollability, and motivated performance. Social-evaluative threat, which results from other people (negatively) judging an individual’s performance, substantially influences the stress response. Tasks involving social-evaluative threat typically challenge core values of the individuals’ self-identity (e.g. intelligence, competence), often well above normal capability. Novel stressors impose demands for which the individual has not been able to develop a coping strategy yet. Many tasks appear less stressful with training. Uncontrollability aims to disrupt previously developed coping strategies (e.g. large speed increase in mental arithmetic tasks). Performance can be motivated by gaining something when accomplishing a task or by losing something in case of failure. However, performance in such tasks must be evaluable (e.g. counting errors in arithmetic tests). All these factors are assumed to work primarily by threatening success, especially when failure affects core values of the social self [20].

### 2.3. Physiological stress response

The human organism responds to acute mental stress mainly via two pathways [1]: the HPA axis and the sympathoadrenal system. The response of the sympathoadrenal system is faster (effects within seconds) than the response of the HPA axis (effects within minutes) [1].

**Fig 2:**
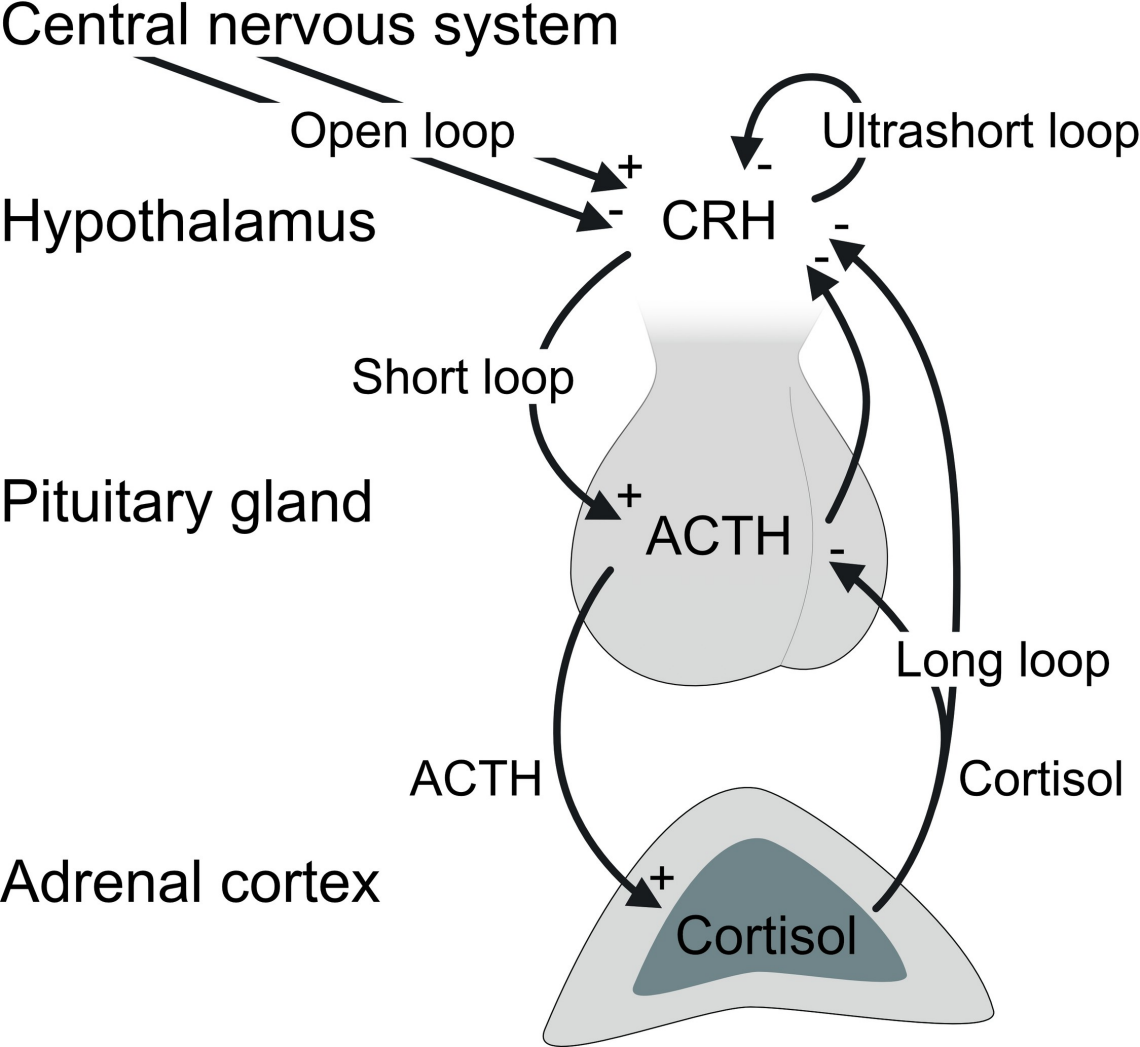
Cascade structure of cortisol secretion in the hypothalamic-pituitary-adrenocortical axis including endocrine feedback mechanisms. Adapted from [47]. ACTH: Adrenocorticotropic hormone. CRH: Corticotropin-releasing hormone.

The *HPA axis* refers to one of the two main pathways involved in the stress response. Its activation causes a cascade of chemical reactions altering the concentration of glucocorticoids as illustrated by Fig 2. Many regions of the central nervous system including the amygdala interact for stress appraisal [46]. The amygdala plays a central role in the integration of information regarding mental stressors, especially those related to adverse affection, causing emotions of fear and anxiety [46]. To elicit a stress response, the amygdala triggers the production of corticotropin-releasing hormone in the hypothalamus, which stimulates secretion of adrenocorticotropic hormone from the pituitary gland into the bloodstream [47]. This excites cortisol production in the adrenal cortex [47]. Increased cortisol enables the body to utilize additional energy sources, more precisely it supports the metabolism by stimulating hepatic glucogenesis as well as the breakdown of tissual protein and fat storages [20]. Cortisol enters saliva by diffusion from blood plasma [48]. Several inhibitory feedback loops to the pituitary gland and the hypothalamus regulate the secretion processes (see Fig 2).

The second main pathway involved in the stress response is the *sympathoadrenal system*. It operates via the neurotransmitters epinephrine (adrenaline) and norepinephrine (noradrenalin), both belonging to the catecholamines [49]. Triggered by the hypothalamus, the brainstem activates efferent sympathetic nerves, which leads to the release of norepinephrine at sympathetic neuroeffector junctions and stimulates the production of epinephrine in the adrenal medulla [49]. The locus coeruleus of the brainstem ensures a coordinated response by providing feedback to cortex, hippocampus, and amygdala [49]. The hormonal actions of epinephrine and the autonomic neuroeffector actions of norepinephrine cause cellular effects in many organ systems (see [49 p. 473]). Effects of sympathetic activation include acceleration of heart rate, increase in myocardial contractility, reduction of heart rate variability, peripheral vasoconstriction as well as vasodilation in skeletal muscles and the heart (centralization of blood flow), acceleration of respiration rate, bronchodilation, increased sweat gland secretion, pupillary dilation, and inhibition of digestive activity [49–51]. Sympathetic activation also influences saliva production in the salivary glands in terms of reduced flow rate and increased protein density, including α-amylase [48]. However, autonomic control of saliva production also heavily depends on parasympathetic activation, which is associated with increased flow rate and reduced protein density [48]. The interplay of both branches of the autonomic nervous system for saliva production is complex and nuanced, making straightforward interpretation of α-amylase difficult [48].

### 2.4. Methods for strain assessment

Clinical and laboratory methods to assess the response to acute mental stress span across three domains: behavioral information, the HPA axis, and the sympathoadrenal system [7–9]. To date, there is no unified standard to quantify acute mental strain [7].

*Behavioral information* is gained by means of psychometric variables from self-report questionnaires which often target the emotional condition to draw inferences about acute mental strain [9]. The multifaceted application of the concept of stress has resulted in a wide variety of questionnaires. The most compact form constitutes single-item Likert scales [52] using descriptors of stress level (e.g. inner tension) or emotions (e.g. happiness, fear, anger, sadness, disgust), as in [53, 54]. A widely adopted variant is the Self-Assessment Manikin, which enquires the three affective dimensions valence, arousal, and dominance with the aid of pictograms on five- or nine-level scales [55, 56]. More differentiated questionnaires include the Perceived Stress Scale [57], the Kessler Psychological Distress Scale [58], the Semantic Differential Scale [59] and the University of Wales Institute of Science and Technology Mood Adjective Checklist (UMACL) [60]. Questionnaires developed for work stress include the Job Content Questionnaire [61], the Effort-Reward Imbalance Questionnaire [62], the Job Stress Questionnaire [63], and the Occupational Stress Questionnaire [64]. As with the methods for stress induction, there are many variations and specialized questionnaires for specific research objectives. For occupational stress, a detailed comparative overview is provided in [65]. However, more complex questionnaires tend to target chronic strain rather than acute strain.

*HPA axis* activation is most commonly studied by means of cortisol concentration [66], which can be acquired from blood, urine, hair, or saliva samples [49]. Saliva appears most suitable as blood sampling requires an invasive method and urine accumulation as well as hair growth take time [49]. The cortisol concentration strongly depends on the diurnal rhythm [49] and typically peaks about 5 – 30 min after stressor onset [1, 9, 20], sometimes even later [66]. Although the cortisol response is not always pronounced, it helped to identify the factors that facilitate acute mental strain, which have already been described in section 3.2 [20].

The *sympathoadrenal system* affects many organs and therefore offers a wide range of options for the assessment of strain. Chemical biomarkers of interest include α-amylase, norepinephrine and norepinephrine spillover rate [9]. Often, alterations of organ functions are investigated such as electrical heart activity, mechanic heart activity, muscular (including vascular) activity, respiration, and sweat secretion. Biosignal acquisition and analysis comprises a range of techniques for the non-invasive study of these organ functions. Well known and frequently utilized techniques for the assessment of acute mental strain are presented in section 3.5. Vital signs of interest derived from biosignals include heart rate and heart rate variability parameters, left ventricular ejection time, stroke volume, cardiac output, pulse transit time, diastolic blood pressure, systolic blood pressure, mean blood pressure, left ventricular work index, blood volume pulsation strength, respiration rate, tidal volume and parameters for phasic and tonic dermal nerve activity [9]. Pupil size, measured with specialized cameras focusing the eyes, has also been utilized [9].

**Fig 3:**
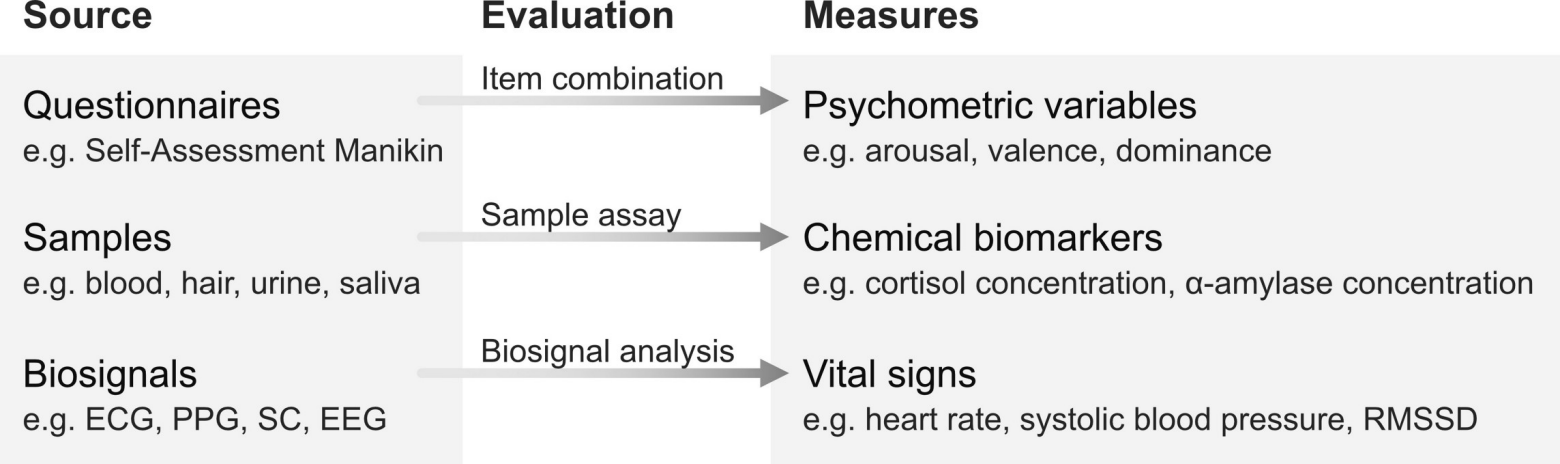
General types of measures for the assessment of acute mental strain.

For clarity, Fig 3 presents a scheme how terms for different types of measures are used in this work.

### 2.5. Biosignals for strain assessment

Besides psychometric variables and chemical biomarkers, monitoring of vital signs is frequently applied to assess both physical and mental strain [9]. Conventional techniques for vital sign monitoring include electrocardiography, photoplethysmography, sphygmomanometry, pneumography, and the measurement of electrodermal activity, which will be briefly introduced in the following. Other techniques, which exceed the scope of this work and for which readers are referred to the literature, include impedance cardiography [67–69], electromyography [70, 71], electroencephalography [72, 73], thermography [74, 75], and electrogastrography [76, 77] as well as the analysis of vocal patterns [78–81], facial expressions [81, 82], and gait [81, 83–85].

Electrocardiography is a technique to record electrical sum potential differences on the surface of the upper body [86]. The electrocardiogram (ECG) allows for non-invasive investigation of the electrical activity of the heart [86]. The most prominent elements of the ECG include P wave (atrial depolarization), QRS complex (ventricular depolarization and atrial repolarization), and T wave (ventricular repolarization) [86]. To examine a series of heartbeats, each beat is localized by the peak of its R wave and the duration between consecutive R waves is called the RR interval [87]. The RR interval series is used to study heart rate variability [87]. The time duration of ventricular excitation reaches from the Q wave to the end of the T wave and is called QT interval [86, 88]. The analysis of a QT interval series allows for the computation of QT variability [88]. As ventricular depolarization appears to be much more stable than ventricular repolarization, QT variability is assumed to reflect mainly fluctuations in ventricular repolarization [88].

Photoplethysmography, extensively described in [89, 90], is an optical technique to track changes in (peripheral) blood volume. A clip with a light source and a photodiode is placed on skin tissue. The amount of light reaching the photodiode fluctuates with the amount of blood in the tissue. Each cardiac contraction initiates a pulse wave traveling through the vascular system. This leads to high-frequent pulsatile fluctuations in the photoplethysmogram (PPG) with the frequency of the heart rate. The technique furthermore allows for the measurement of peripheral vasomotion (vasodilation, vasoconstriction) as the sympathetic tone on vascular smooth muscles alters the amount of blood on a low-frequent level. Multispectral photoplethysmography allows for pulse oximetry, the non-invasive measurement of blood oxygen saturation [89, 90].

Sphygmomanometry, summarized in [91], comprises a range of techniques for cuff-based intermittent blood pressure measurement. The cuff is placed on the upper arm at heart level and inflated above systolic blood pressure to occlude all vessels. The cuff then deflates but blood can flow only when brachial blood pressure exceeds cuff pressure. The moment this occurs the first time marks the systolic arterial blood pressure. Blood flow is turbulent in the beginning and becomes laminar when the cuff pressure is too small to affect the profile of the brachial artery. This marks the diastolic arterial pressure. Start and end of turbulent blood flow can be detected by means of auscultatory, palpatory, and oscillometric measurement [91]. Non-invasive continuous blood pressure measurement utilizes photoplethysmography to control the pressure of a small cuff placed at a finger [92, 93]. In- and deflation of the cuff are tuned to compensate the cardiac pulsation component in the PPG [92]. However, continuous measurements require calibration with intermittent measurements to model arterial blood pressure [91, 93].

Pneumography comprises a range of techniques for measurements regarding the lungs. Techniques vary widely in complexity and capabilities [94]. Abdominal and thoracic straps with resistive strain gauges suffice for the assessment of breath rate due to changes in abdominal and thoracic expansion during breathing [94]. Inductive pneumography utilizes transducers in the straps to measure changes in self-induction due to abdominal and thoracic volume changes [95]. Thermistors are used to detect nasal or oronasal airflow [96] and the thoracic impedance can be measured with a high-frequent current between two or more electrodes [97]. As respiration modulates heart rate (respiratory sinus arrhythmia), breath rate can be measured indirectly from ECG and PPG [98]. More detailed information about volumes, pressures, or chemical compositions require spirometry or capnography [94].

Electrodermal activity, about which a comprehensive work can be found in [99], is a technique to measure the electrical properties of the skin surface. Most often, a small direct current is sent between two electrodes placed in close proximity (e.g. on the thenar and hypothenar eminences of the hand) to measure the conductance of the skin. Skin conductance mainly depends on sweat secretion, which is exclusively controlled by sympathetic excitation. Electrodermal activity comprises low-frequent tonic and high-frequent phasic components. While the tonic component reflects the overall electrodermal level, the phasic component covers specific electrodermal reactions of a few hundred milliseconds occurring in response to stimulation or spontaneously [99].

### 2.6. Summary and study motivation

Stress is a multifaceted topic and its investigation requires interdisciplinary competence from the fields of psychology, physiology, and biomedical engineering. ISO 10075-1 provides a standardized definition of acute mental stress and strain, which is an essential prerequisite for interdisciplinary research. The collection of clinical and laboratory methods for stress induction illustrates the diversity of approaches. Even though strain assessment typically assumes mediation by the HPA axis and the sympathoadrenal system, measurement methods and parameters vary widely, which introduces a certain degree of randomness into the selection of vital signs for the assessment of acute mental strain.

To reconcile this variety and focus on the most important parameters for effective strain assessment, we conducted the experimental study presented in the following chapters to answer four research questions (RQ). RQ1: Which biosignal vital signs change in the acute mental stress experiment? Hypothesis: All acquired vital signs that measure the electrical heart activity, cardiovascular haemodynamics, skin conductance, and respiration change over the course of the experiment. RQ2: Which biosignal vital signs show immediate changes in response to acute mental stress compared to baseline? Hypothesis: Acute mental stressful stimulation leads to immediate changes of the baseline values of all acquired vital signs that measure the electrical heart activity, cardiovascular haemodynamics, skin conductance, and respiration. RQ3: If a reduced number of biosignal vital signs had to be selected, what subset of vital signs allows effective detection of stressful stimulation in differentiation from baseline and recovery? Hypothesis: A subset of important vital signs from all acquired vital signs suffices to effectively detect when acute mental stress is induced. RQ4: How do the chemical biomarkers cortisol concentration and α-amylase concentration react in response to acute mental stress? Hypothesis: Cortisol concentration and α-amylase concentration increase with a delay of a few minutes after the beginning of acute mental stress induction and turn towards recovery within 45 min after acute mental stress induction ended.

## 3. Materials and methods

To investigate the human response to acute mental stress, the experimental study described in the following was set up. The study was approved by the Ethics Committee of the TU Dresden (Office for Human Research Protections registration codes IRB00001473, IORG0001076) under the reference number EK411092019.

### 3.1. Participants

We acquired healthy participants on a voluntary basis with a small remuneration of 10 € for 2 hours in the period January 2021 to December 2022. Participants had to meet the following eligibility criteria:

- age range 18–40 years,
- no obesity or underweight,
- no known cardiovascular diseases,
- no known neurological disorders,
- no known mental disorders,
- no known endocrinological disorders,
- no known acute allergies,
- no medication or drug use,
- no known pregnancy.

In total, 65 participants (33 female, 32 male) took part in the study. Issues during data collection led to the exclusion of 5 recordings so that the final examination contained data from 60 participants. Table 2 presents the main characteristics of this participant group. Identification of participants after data collection was prevented by subject coding (no follow-ups intended), but information to match subject coding and participant identification is possible upon special request (see Data Availability statement).

**Table 2:** Descriptive statistics for the demographic data. The participant group consisted of 60 people (28 female, 32 male).

	Age	Weight	Height	Body Mass Index
	in years	in kg	in cm	in kg/m²
Mean ± SD	25.8 ± 5.1	68.3 ± 12.4	174.7 ± 9.6	22.3 ± 2.7
Range	18 − 40	50 − 108	155 − 194	17.4 − 31.6

### 3.2. Stimulus

To induce acute mental stress, we utilized the Mannheim Multicomponent Stress Test (MMST) developed and presented in [24, 25] for a period of 5 min. We chose this method because it combines several mental stressors to maximize acute mental strain while refraining from social-evaluative stressors (see section 3.2) [24]. The main element of the MMST is cognitive load induced by a simple arithmetic task (computerized version of Paced Auditory Serial Addition Task). Participants must respond quickly while gradually swelling white noise and affective images are played in the background. Response time shortens from 3 s to 2 s in the middle of the test to incorporate uncontrollability. Erroneous or missing answers trigger a disruptive sound to incorporate motivated performance. Participants had to maximize their performance as they were told that they lose a part of their remuneration money with each erroneous or missing answer. A video showing the test screen of the MMST is provided in S1 Video.

The MMST was preceded by a training period (1 min) without white noise and affective pictures to ensure participants understood the main task and familiarized with the user interface. All instructions for the MMST including introduction and training were given in written format via the stimulus monitor.

### 3.3. Experimental procedure

As recommended by Dickerson and Kemeny [20], all trials were conducted in the late afternoon due to the circadian rhythm of cortisol concentration. This is beneficial also for the measurement of other vital signs, e.g. those related to heart rate variability and QT variability, as they are influenced by circadian rhythm, which follows cardiac autonomic tone [88]. We offered two slots starting at 3:00 PM and 5:30 PM (stressor onset approx. 45 – 50 min after start). Participants were instructed to refrain from the consumption of nicotine, alcohol, and drugs as well as caffeine (4 h), and large meals (3 h) prior to the trial.

Participants were made familiar with the temporal structure of the trial, the modalities of measurement and the concept of the arithmetic task. After clarification of data privacy and given written consent, participant information was collected. This included demographic data (age, height, weight), a control of the consumption instructions and eligibility criteria, the Perceived Stress Questionnaire (PSQ) [100] (German version [101, 102]) to evaluate the mid- to long-term psychosomatic strain of the last four weeks, and self-assessment of the skin type in accordance with official methods of the German Federal Office for Radiation Protection [103] and the Australian Radiation Protection and Nuclear Safety Agency [104]. This introductory phase lasted about 20 min and served as acclimatization time. In the next step, the measurement equipment was attached. After application of electrodes and sensors, participants were asked to take a comfortable position, to rest their head on an individually adjusted head-neck support, and to remain still during the rest and stress phases of the trial. Then, participants rested undisturbed while the setup was tested and calibrated. Attachment of the measurement equipment, technical testing, and calibration took about 20 min.

**Fig 4:**
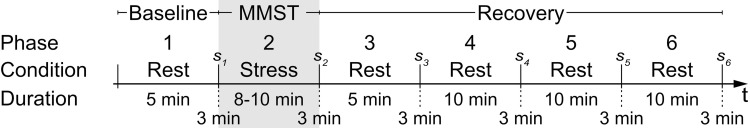
Temporal structure of a trial. Self-assessment was queried and saliva samples were taken after each phase (*s*_1_-*s*_6_).

Fig 4 summarizes the procedural structure of a trial. Trials started with a phase of 5 min baseline recording during rest, followed by the MMST in the second phase. To observe the development of the chemical biomarkers, four phases of rest followed the MMST (1 x 5 min, 3 x 10 min). During all rest phases a relaxation video of a solitary beach bay [105] was played. Phases lasted at least 5 min to meet the requirements for short-term heart rate variability measurements [87] and were extended to 10 min to adapt typical saliva sampling intervals [20, 25]. After each of the six phases, a saliva sample was collected with the Salivette Cortisol (Sarstedt AG & Co. KG, Nümbrecht, Germany) while the participants filled out a self-assessment questionnaire about the currently perceived subjective stress level. To stimulate saliva production, participants were instructed to agitate the mandible without chewing or moving the absorbent roll around (2 min). The self-assessment consisted of a ten-level Likert scale (SAL, “unstressed” to “very stressed”) as well as a five-level Self-Assessment Manikin (SAM, emotional dimensions valence, arousal, and dominance [106]) questionnaire. Saliva sampling and self-assessment together required about 3 min. During this time, the finger for continuous blood pressure measurement was alternated to reduce physical strain on the fingers and recalibrate.

Trials were conducted in seated position with a head-neck support to reduce head movements. Participants sat quiet and alone from the moment instructions were given after the application of the measurement equipment. Interaction with the testing personnel was restricted to the collection of the saliva samples.

### 3.4. Data acquisition

**Fig 5:**
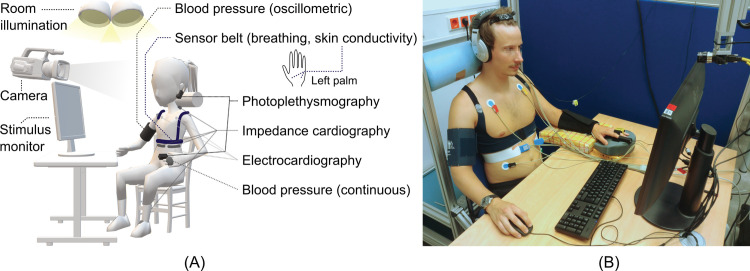
Experimental setup. (A) Labeled schematic illustration of sensor application. (B) Photo of a participant in the setup (re-staged after trial completion, image rights granted).

Three technical systems were combined for multimodal data acquisition during the trial. System 1 comprised devices from ADInstruments Ltd. (Dunedin, New Zealand). System 2 consisted of a Task Force Monitor 3040i from CNSystems Medizintechnik GmbH (Graz, Austria), and system 3 covered an industrial camera UI-3060CP-C-HQ Rev.2 from IDS Imaging Development Systems GmbH (Obersulm, Germany). Table 3 provides an overview over acquired biosignals and their recording specifications. Fig 5 illustrates the sensor application and shows an exemplary photo of a participant in the experimental setup. The distance between camera and face amounted to approximately 0.7 m. Daylight was blocked from the laboratory by roller shutters so that only the ceiling lights illuminated the scene (44 fluorescent tubes, Osram Lumilux L 18 W/840).

**Table 3:** Overview over the biosignals recorded and the hardware utilized.

System	Device	Signal	Location	Specification
1	MLT1020FC	Photoplethysmogram	Finger (left index)	1000 Hz, 950 nm
	MLT1020EC	Photoplethysmogram	Earlobe (left)	1000 Hz, 950 nm
	Equivital sensor belt	Electrocardiogram	Chest	256 Hz, two non-standard leads
	Equivital sensor belt	Chest expansion	Thorax	25.6 Hz, resistive strain gauge
	Equivital GSR expansion	Skin conductance	Hand (left, thenar and hypothenar eminences)	16 Hz, direct current
2	Task Force Monitor 3040i	Blood pressure (continuous)	Finger (alternating left middle and ring)	100 Hz, vascular unloading technique
	Task Force Monitor 3040i	Blood pressure (intermittent)	Brachial (right)	Oscillometric
	Task Force Monitor 3040i	Electrocardiogram	Chest	1000 Hz, Einthoven leads I and II
	Task Force Monitor 3040i	Impedancecardiogram	Chest	500 Hz
3	Camera UI-3060CP-C-HQ Rev.2 with lens CINEGON 1.8/16-0910	RGB video	Face and shoulders	100 fps, 12 bit, 320 x 640 px, uncompressed

To evaluate the signals of the different systems together, the systems must be synchronized. System 1 provided the reference time. This system operated with the data acquisition hardware PowerLab 16/35 in combination with the ADInstruments software LabChart 8. The Equivital sensor belt, also part of system 1, transmitted data via Bluetooth directly to LabChart and was synchronized automatically. The camera of system 3 sent an analogue frame trigger signal to the PowerLab, which sampled the frame trigger signal with 1000 Hz and therefore allowed temporal alignment of system 3 to system 1. To synchronize system 2, the PowerLab generated an analogue synchronization signal (changing basic functions like rectangle or triangle) that was sampled with 1000 Hz by both the PowerLab itself and the Task Force Monitor. The congruent superposition of these two signals allowed precise temporal mapping of system 2 to system 1. Compensation of slightly differing sample rates by time stretching has already been described in a preliminary analysis [107]. All data processing was performed using MATLAB (The MathWorks, Inc., Natick, MA, USA).

### 3.5. Signal processing

ECG signals were filtered by using a digital Butterworth high-pass filter (filter order 30, cut-off frequency 0.3 Hz) [108]. To extract RR intervals, QT intervals, and T wave amplitudes from ECG, an iterative implementation of the two-dimensional signal warping (i2DSW) algorithm [108, 109] was applied. i2DSW uses a template approach to robustly estimate time intervals on a beat-to-beat basis. Beat templates were automatically generated and underwent a manual review by an expert to be excluded from further analysis if necessary. Automatic beat rejection [108] and RR filtering [110] were applied to exclude noisy heart beats and abnormal RR intervals (e.g. extrasystoles). Extracted RR series and QT series were excluded from further analyses if more than 50% of RR or QT intervals had been rejected or filtered.

A cascade of high- and low-pass filters extracted the cardiac pulse signal from the earlobe PPG and the finger PPG (5^th^ order Butterworth, cut-off frequencies 0.5 Hz and 5 Hz). For the earlobe PPG, also the low-frequent signal component was extracted (5^th^ order Butterworth low-pass filter, cut-off frequency 0.5 Hz). All filters used the zero-phase approach to preserve synchronicity, i.e. signals passed filters one time forward and one time reversed so that the phase shifts resulting from the two opposed filter operations canceled each other out. We utilized the PhysioNet Cardiovascular Signal Toolbox (version 1.0.2) [111] to calculate pulse onset annotations.

All annotations (R peak in ECG, pulse onset in earlobe PPG, and pulse onset in finger PPG) underwent the semi-automated validation procedure for verification described in [107]: Based on a signal quality analysis, suspicious signal segments and annotations were identified. All annotations within suspicious signal segments underwent manual review by an expert [107].

Skin conductance measurements, recorded between the thenar and hypothenar eminences (left hand) as recommended for the exosomatic direct current method [99], were processed with the toolbox Ledalab (version 3.49) [112]. Preprocessing to denoise the signals consisted of a 5^th^ order low-pass filter with cut-off frequency 0.16 Hz. To split tonic and phasic activity, we applied the Continuous Decomposition Analysis method [113] with an amplitude threshold of 0.05 μS for phasic responses [114] and the maximum of eight iterations to optimize the impulse response function for each participant.

Respiration signals were gained directly from chest expansion without pre-processing.

### 3.6. Vital sign extraction

In total, we extracted 60 vital signs related to heart rate variability, QT variability, haemodynamic properties, skin conductance, and respiration from the biosignals as well as the two chemical biomarkers cortisol and α-amylase concentration from the saliva samples.

Table 4 provides a description for the vital signs derived from biosignals. Parameters measured multiple times within the 5 min period were statistically summarized by their median value if not stated otherwise.

**Table 4:** Overview over the vital signs extracted from biosignals in this study. Description of heart rate variability measures adopted from [115]. Description of QT variability measures according to [88, 119]. Description of haemodynamic measures apart from PATear, PATfinger, and DC according to [120]. BSA: Body surface area.

Parameter	Unit	Description
**Heart rate variability measures**		
Time domain		
RRmean	ms	Mean duration of RR intervals
SDRR	ms	Standard deviation of RR intervals
RRVN	-	Squared coefficient of variation of RR intervals
STVRR	ms	Short-term RR interval variability
RMSSD	ms	Root mean square of successive RR interval differences
SDSD	ms	standard deviation of successive RR interval differences
pNN50	-	Proportion of successive RR intervals that differ by more than 50 ms
NN50	-	Number of successive RR intervals that differ by more than 50 ms
TRI	-	Triangular index (integral of the density of the RR interval histogram divided by its height)
TINN	ms	Baseline width of the RR interval histogram
Frequency domain		
VLF	s^2^	Absolute power of the very-low-frequency band (0.0033 Hz – 0.04 Hz)
LF	s^2^	Absolute power of the low-frequency band (0.04 Hz – 0.15 Hz)
HF	s^2^	Absolute power of the high-frequency band (0.15 Hz – 0.4 Hz)
LFHFratio	-	Ratio of LF-to-HF power
LFn	-	Relative power of the low-frequency band (0.04 Hz – 0.15 Hz) in normal units
HFn	-	Relative power of the high-frequency band (0.15 Hz – 0.4 Hz) in normal units
Non-linear		
ApEn	-	Approximate entropy, which measures the regularity and complexity of a time series
DFA1	-	Detrended fluctuation analysis α1, which describes short-term fluctuations
DFA2	-	Detrended fluctuation analysis α2, which describes long-term fluctuations
SD1	ms	Poincaré plot standard deviation perpendicular to the line of identity
SD2	ms	Poincaré plot standard deviation along the line of identity
SD1SD2ratio	-	Ratio of SD1-to-SD2
**QT variability measures**		
QT interval and T wave		
QTmean	ms	Mean duration of QT intervals
QTc_(Bazett)_	ms	Bazett’s corrected QT interval
QTc_(Fridericia)_	ms	Fridericia’s corrected QT interval
Tamp	μV	Mean T wave amplitude
QT variability		
SDQT	ms	Standard deviation of QT intervals
cSDQT	-	T wave amplitude-corrected standard deviation of QT intervals
QTVN	-	Squared coefficient of variation of QT intervals
STVQT	ms	Short-term QT variability
LTVQT	ms	Long-term QT variability
QT variability normalized to heart rate variability		
QTVi	-	QT variability index
cQTVi	-	T wave amplitude-corrected QT variability index
QTRRslope	-	Linear slope between RR and QT intervals
VR	-	Variability ratio of STVQT and STVRR
**Haemodynamic measures**		
SV	ml	Stroke volume
SI	ml/m^2^	Stroke index (SV normed to BSA)
CO	l/min	Cardiac output
CI	l/(min*m^2^)	Cardiac index (CO normed to BSA)
TPR	dyn*s/cm^5^	Total peripheral resistance
TPRI	dyn*s*m^2^/cm^5^	Total peripheral resistance index (TPR normed to BSA)
dBP	mmHg	Diastolic blood pressure
mBP	mmHg	Mean blood pressure
sBP	mmHg	Systolic blood pressure
ppBP	mmHg	Pulse pressure (difference between sBP and dBP)
ACI	100/s^2^	Acceleration index
EDI	ml/m^2^	End diastolic index (preload, end-diastolic volume of the left ventricle normed to BSA)
IC	1000/s	Index of contractility (estimation of maximum blood flow during the left ventricular ejection)
LVET	ms	Left ventricular ejection time
LVWI	mmHg*l/(min*m^2^)	Left ventricular work index
TFC	1/kΩ	Thoracic fluid content
PATear	ms	Pulse arrival time to earlobe (time delay between R peak in ECG and pulse onset in earlobe PPG)
PATfinger	ms	Pulse arrival time to finger (time delay between R peak in ECG and pulse onset in finger PPG)
DC	-	Average low-frequent component intensity of earlobe PPG
**Skin conductance measures**		
SCL	μS	Skin conductance level (tonic activity)
NSCRpm	1/min	Number of phasic skin conductance responses per minute
SCRamp	μS	Amplitude of phasic skin conductance responses
SCRriseTime	s	Time from onset to peak of phasic skin conductance responses
**Respiration measures**		
BR	rpm	Breath rate
BRV	rpm	Breath rate variability (standard deviation of BR)

Heart rate variability measures originated from the RR interval time series with segments of 5 min length to meet the recommendations for short-term measurements [87, 115]. Heart rate variability measures can be categorized into three domains: time domain, frequency domain and non-linear [87]. We calculated the most common heart rate variability measures in accordance with [87]: average normal-to-normal beat (RRmean), standard deviation of RR intervals (SDRR), standard deviation of successive RR interval differences (SDSD), power in high-frequency (HF), low-frequency (LF), and very low-frequency band (VLF), and approximate entropy (ApEn) next to detrended fluctuation analysis measures to quantify non-linear relations. The complete overview of the calculated heart rate variability measures is given in Table 4.

Consistent with heart rate variability measures, QT variability measures were calculated from QT interval time series of 5 minutes in length [88]. For each 5-min segment, the following measures were calculated: average QT interval (Qtmean), the rate-corrected QT interval using Bazett’s QTc_(Bazett)_ [116] and Fridericia’s formula QTc_(Fridericia)_ [117], standard deviation of QT intervals (SDQT), and the QT variability index (QTVi) [118] quantifying the relation between QT variability and heart rate variability. To account for the inverse relationship between QT variability and T wave amplitude (Tamp), T wave amplitude-corrected SDQT (cSDQT) and QTVi (cQTVi) were calculated [119]. Besides these, the most common parameters of QT variability [88] were calculated to allow systematic comparison, see Table 4.

Haemodynamic measures (apart from the parameters PATear, PATfinger, and DC, see Table 4) originated from the Task Force Monitor, which provided information on a beat-to-beat basis. This includes stroke volume (SV), cardiac output (CO) as well as diastolic (dBP), systolic (sBP), and mean (mBP) blood pressure. Extraction techniques of the Task Force Monitor are described in [120]. The complete overview over haemodynamic measures is given in Table 4. PATear, PATfinger, and DC were measured over segments of 10 s length. Pulse arrival times PATear and PATfinger were derived from the validated annotations as the time delay between R peak in the ECG and pulse onset in the earlobe or finger PPG, respectively, as described in [107]. The mean intensity of the low-frequent component of the earlobe PPG yielded the parameter DC [89].

Skin conductivity measures originated from the Ledalab toolbox for the full length of each phase. Ledalab provided the mean skin conductance level (SCL) to investigate tonic activity and the number of phasic skin conductance responses per minute (NSCRpm), their mean amplitude (SCRamp) as well as their mean time from onset to peak (SCRriseTime) to investigate phasic activity.

Respiration measures originated from the chest expansion signal. Following the description in [121], spectral analysis of segments of 30 s length yielded breath rates (BR). Breath rate variability (BRV) marks the standard deviation of the breath rates measured over the course of 5 min.

A specialized laboratory (Dresden LabService GmbH, Dresden, Germany) performed assays to gain cortisol concentrations *c*_*cort*_ and α-amylase concentrations *c*_*amyl*_ from the collected saliva samples.

### 3.7. Statistical evaluation

Statistical evaluation was performed with MATLAB R2022b unless stated otherwise.

RQ1: To check for differences across the six phases (see section 3.6), we performed a repeated-measures analysis of variance (RMANOVA) for each biosignal vital sign. To protect the RMANOVA from outlier distortion, vital signs were filtered with the MATLAB function rmoutliers in its standard configuration. We tested sphericity with Mauchly’s test and applied the Greenhouse-Geisser correction in case of violated sphericity. The significance level *α* = 0.05 for the RMANOVAs was adjusted by Bonferroni correction.

RQ2: To investigate the immediate response to acute mental stress, we used a two-sample t-test (two-tailed) for each biosignal vital sign to check for differences between the baseline rest phase and the MMST phase. The significance level *α* = 0.05 for the two-sample t-tests was adjusted by Bonferroni correction.

RQ3: To identify the most important vital signs for effective detection of stressful stimulation within a single model, we utilized a binary logistic regression with the forward selection method (conditional) from SPSS Statistics 28.0 (IBM Corporation, Armonk, NY, USA). In this context, the condition was predicted, i.e. acute mental stress for phase 2 and rest for the five other phases covering baseline and recovery (see Fig 4), from a subset of all 60 available biosignal vital signs. The subset was altered by stepwise inclusion and exclusion of vital signs in a statistical optimization approach, see [122].

RQ4: The evaluation of salivary cortisol concentration followed the procedure applied by Dickerson and Kemeny [20] by calculating the effect size *d*_*cort*_ for each sampling point *s*_*i*_:

$$d_{cort}(s_{i})=\frac{{\bar{c}}_{cort}(s_{i})-{\bar{c}}_{cort}(s_{1})}{\text{SD}(s_{i})}=\frac{\left(\frac{1}{N_{p}}\sum_{j=1}^{N_{p}}c_{cort}(p_{j},s_{i}))-(\frac{1}{N_{p}}\sum_{j=1}^{N_{p}}c_{cort}(p_{j},s_{1})\right)}{\sqrt{\frac{1}{N_{p-1}}\sum_{j=1}^{N_{p}}(c_{cort}(p_{j},s_{1})-(\frac{1}{N_{p}}\sum_{j=1}^{N_{p}}c_{cort}(p_{j},s_{1}))}{)}^{2}}$$ (1)

with trial phase *i* ∈ {1,2,…,6} (see Fig 4), and *p*_*j*_ denoting participant *j* ∈ {1,2,…,*N*_*p*_} with *N*_*p*_ representing the total number of participants in the study. To compensate for the changes induced by the diurnal rhythm, intra-individual detrending was applied before calculation of *d*_*cort*_. For the sake of systematics, salivary α-amylase concentrations were evaluated in the same manner as salivary cortisol concentrations to gain the effect sizes *d*_*amyl*_(*s*_*i*_).

## 4. Results

**Fig 6:**
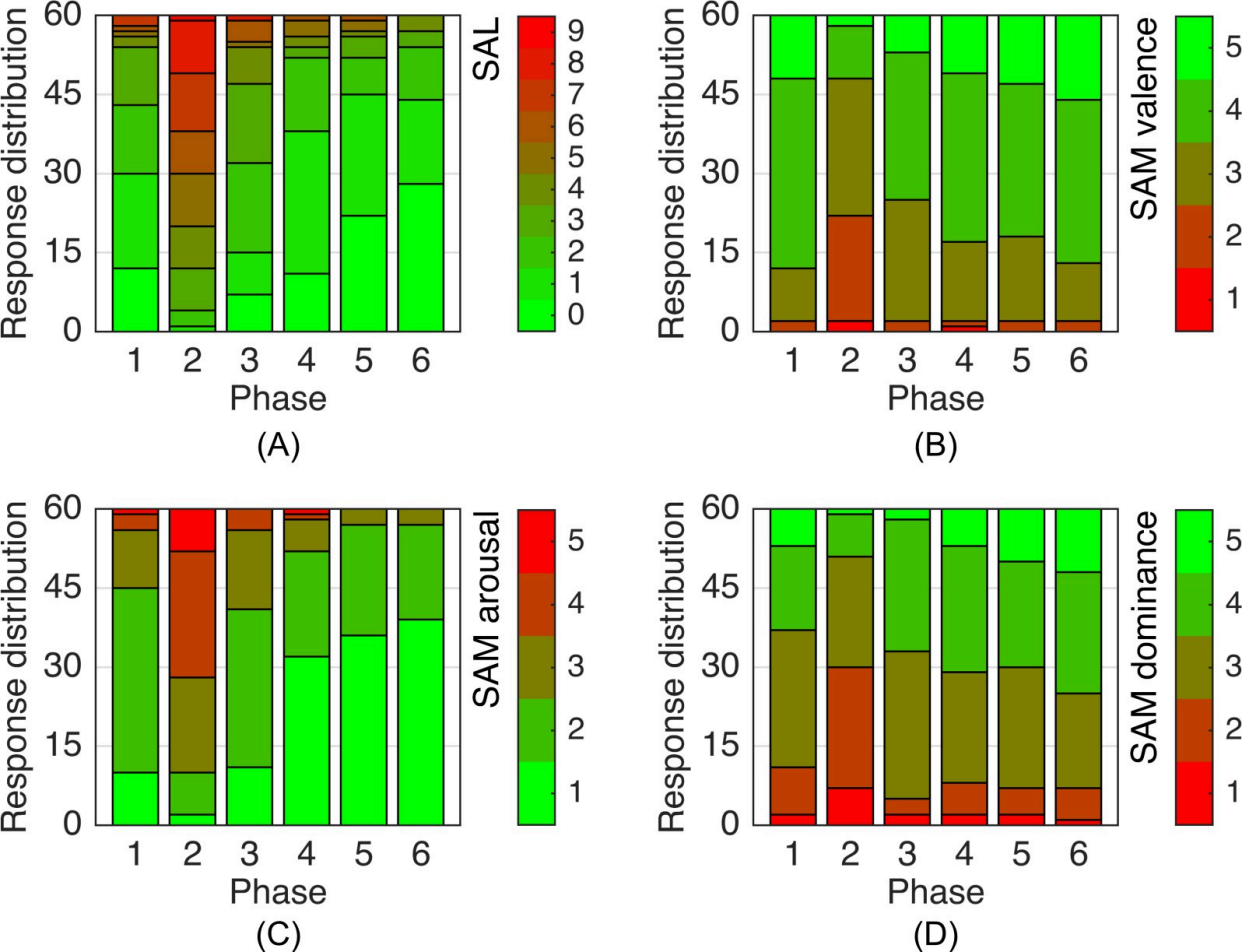
Results of the self-assessment measures. Response distribution of the 60 participants in the form of stacked bar plots for the self-assessment after each phase. Acute mental stress induced in phase 2. SAM: Self-Assessment Manikin. (A) Likert scale (0: unstressed, 9: very stressed). (B) SAM valence. (C) SAM arousal. (D) SAM dominance.

The PSQ score of the participants amounted to 20.2 ± 9.7 (mean ± standard deviation). Only two participants reached PSQ scores larger than 40 (45 and 51). Fig 6 contains the results of the psychometric variables from the self-assessment after each of the six phases. In comparison to the baseline rest phase, SAL and SAM arousal increased during the acute mental stress phase by +197% and +60% (change of mean), respectively, while SAM valence and SAM dominance decreased by -29% and -22%, respectively. Changes between baseline rest phase and acute mental stress phase were statistically highly significant for all self-assessment measures (*p* < 10^−6^, two-tailed Wilcoxon signed-rank test).

A summary of the biosignal vital sign data across all phases and statistical testing results of the RMANOVA (RQ1) are provided in S1 Table. Of the 60 vital signs, 46 exhibited significant differences in the RMANOVA. The 14 vital signs unaffected over time belonged to the heart rate variability measures (SDRR, RRVN, STVRR, tri, TINN, LF, DFA1, SD2), the QT variability measures (cSDQT), and the haemodynamic measures related to the mechanics of systolic left ventricular constriction (SV, SI, EDI, IC, LVET).

**Fig 7:**
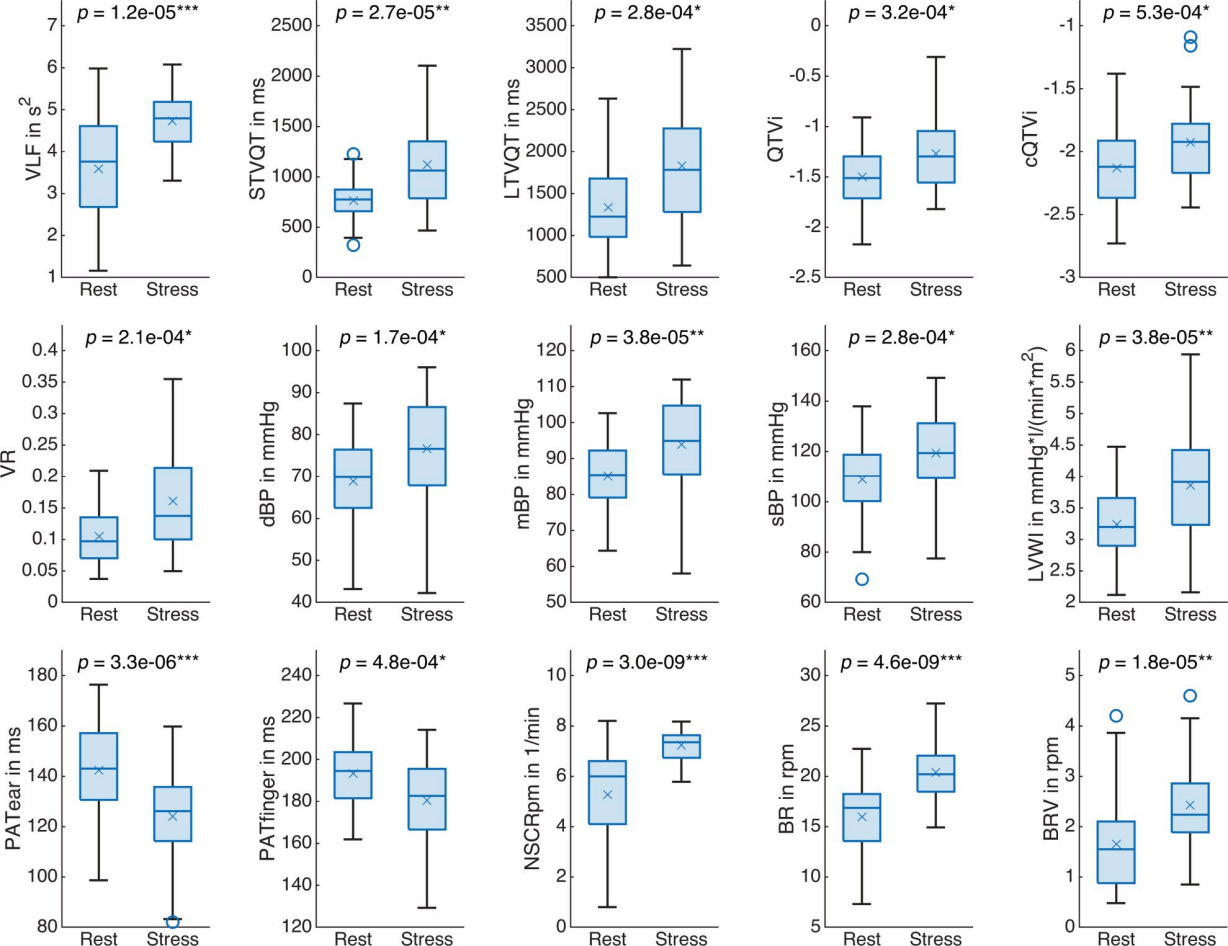
Boxplots for the baseline rest phase and the acute mental stress phase for vital signs with significant t-test results. *P*-value from two-sample t-test. Bonferroni-corrected significance levels indicated with *: *p* < 0.05/60, **: *p* < 0.01/60, ***: *p* < 0.001/60. ×: Mean value.

**Fig 8:**
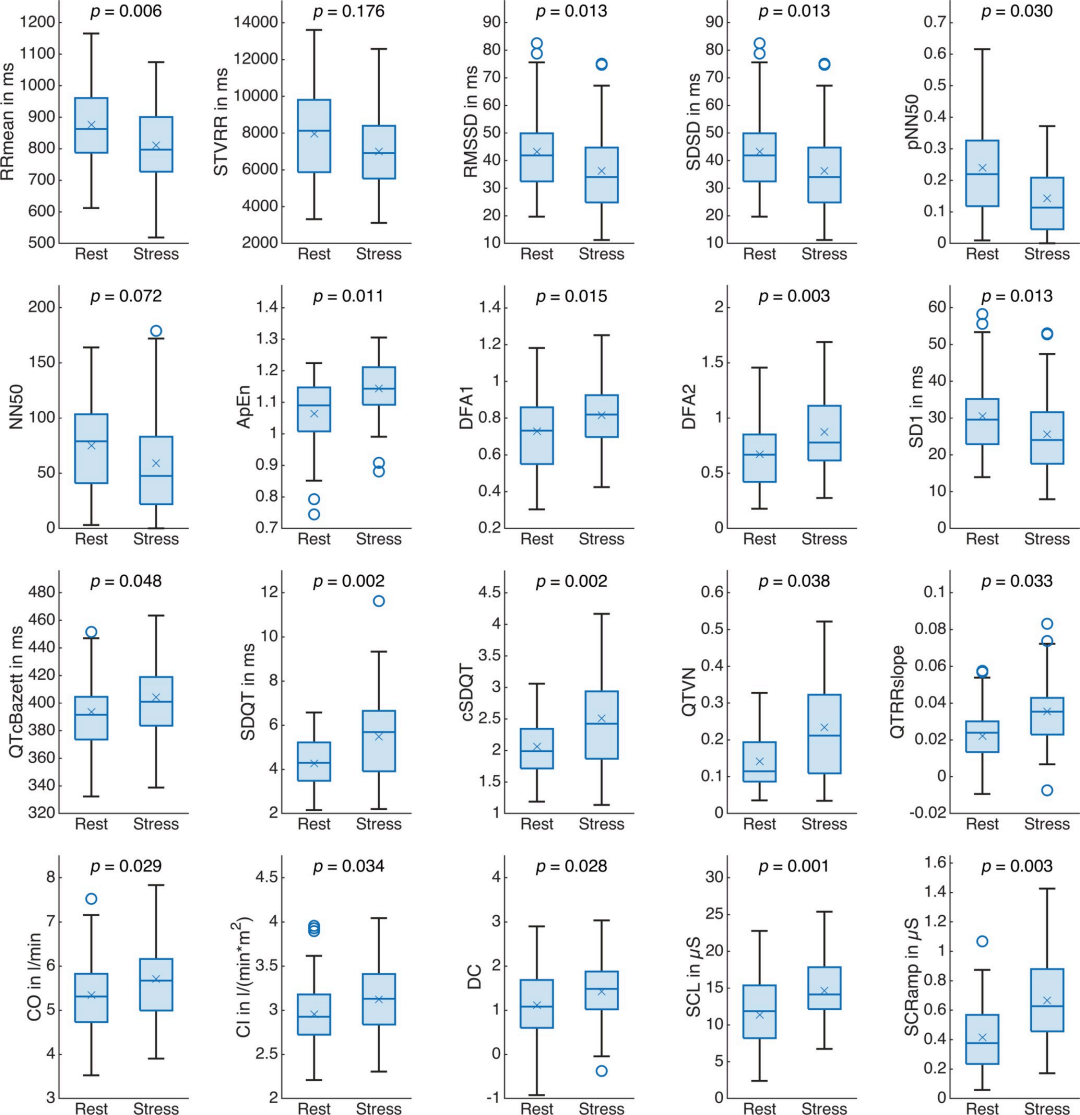
Boxplots for the baseline rest phase and the acute mental stress phase for vital signs without significant t-test results that showed a trend. *p*-value from two-sample t-test. ×: Mean value.

Fifteen of the 60 vital signs yielded significant results in the two-sample t-test (RQ2): VLF, STVQT, LTVQT, QTVi, cQTVi, VR, dBP, mBP, sBP, LVWI, PATear, PATfinger, NSCRpm, BR, and BRV. Fig 7 illustrates the responses of these vital signs to acute mental stress. PATear and PATfinger decreased due to acute mental strain while all other significantly changing vital signs increased in comparison to the baseline rest phase. Fig 8 extends Fig 7 for vital signs that did not yield significant results in the two-sample t-test but exhibited a trend. A similar illustration for all other parameters is provided in S1 Fig.

The vital sign selection approach for the logistic regression (RQ3) converged (p > 0.001, see Table B in S1 Appendix) after identifying the following eight vital signs for the prediction of acute mental stress: QTVi, LVWI, PATear, SCL, NSCRpm, SCRriseTime, BR, and BRV (see Table 5). This model reached a sensitivity of 78.0%, a specificity of 97.6%, and an F1 score of 0.82 (Cox and Snell R^2^ ∈ [0, 0.75]: 0.665, Nagelkerke R^2^ ∈ [0, 1]: 0.886). Detailed statistics for the vital sign selection with the logistic regression approach are provided in S1 Appendix.

**Table 5:** Results reported by SPSS for the final iteration of the logistic regression approach. B: Regression coefficient. C.I.: Confidence interval. df: Degree of freedom. exp(B): Estimated odds ratio. S.E.: Standard error of B.

Vital sign	B	S.E.	Wald	df	*p*	exp(B)	95% C.I. for exp(B)	
							Lower	Upper
QTVi	3.237	1.053	9.449	1	0.002	25.447	3.231	200.408
LVWI	1.344	0.616	4.764	1	0.029	3.834	1.147	12.816
PATear	-0.079	0.019	18.061	1	0.000	0.924	0.891	0.958
SCL	-0.305	0.091	11.181	1	0.001	0.737	0.616	0.881
NSCRpm	1.672	0.406	16.937	1	0.000	5.321	2.400	11.796
SCRriseTime	-2.985	0.918	10.564	1	0.001	0.051	0.008	0.306
BR	0.384	0.108	12.703	1	0.000	1.469	1.189	1.814
BRV	0.780	0.372	4.386	1	0.036	2.181	1.051	4.525

**Fig 9:**
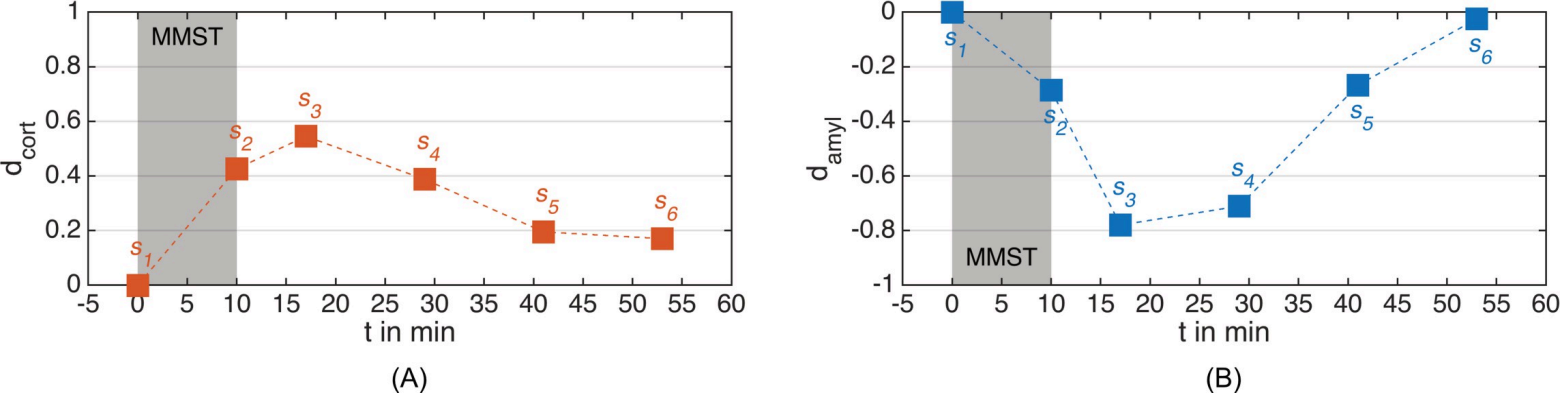
Results on the effect sizes of the salivary concentrations. MMST: Mannheim Multicomponent Stress Test. *s*_*i*_: Sampling point after block *i* (see Fig 4). (A) Salivary cortisol concentration. (B) Salivary α-amylase concentration.

Fig 9 contains the results for the chemical biomarkers (RQ4). Salivary cortisol and α-amylase concentrations peaked approx. 18 min after stressor onset. While cortisol concentrations increased (*d*_*cort*_(*s*_3_) = 0.55), α-amylase concentrations decreased (*d*_*amyl*_(*s*_3_) = -0.78) in response to acute mental stress. Both chemical biomarkers mostly recovered within the trial duration.

## 5. Discussion

The MMST was developed in 2010 by Kolotylova *et al*. [24] under investigation of cardiovascular vital signs. It was validated by means of salivary cortisol concentration and skin conductance measures in 2012 [25]. Two other studies researching schizophrenia utilized the test and reported vital signs for healthy participants from the control group [123, 124]. Table 6 summarizes the results from the literature in direct comparison to our results. In the following, we discuss our results in detail against the state of the art to answer RQ1 and RQ2. In general, there is large agreement in both magnitude and direction of the stress responses; only QT variability measures indicate contradictory results. However, our results on QT variability are consistent with physiological reasoning on the background of sympathetic excitation in response to acute mental stress.

**Table 6:** Comparison of results from the literature reported for the Mannheim Multicomponent Stress Test (MMST). ΔM marks the relative change of the mean values from baseline to the MMST.

Parameter	Unit	Source	Results from literature			Our results		
			Baseline	MMST	ΔM	Baseline	MMST	ΔM
SAL	-	[24]	0.7 ± 0.8	7.1 ± 1.8	+914%	1.8 ± 1.6	5.5 ± 2.0	+206%
"	"	[25]	1.0 ± 1.1	7.1 ± 1.7	+610%	"	"	"
RRmean	ms	[24]	875 ± 112*	800 ± 118*	-9%	885 ± 138	811 ± 129	-8%
"	"	[25]	788 ± 131*	636 ± 113*^†^	-19%	"	"	"
"	"	[123]	822 ± 127*	723 ± 145*	-12%	"	"	"
"	"	[124]	845*^§^	732*^§^	-13%	"	"	"
RRVN	-	[123]	4.58 ± 4.41	4.07 ± 4.08	-11%	5.9 ± 5.6	5.3 ± 3.3	-10%
RMSSD	ms	[24]	45.39 ± 17.28	38.3 ± 16.14	-16%	46.0 ± 18.2	37.3 ± 16.1	-19%
"	"	[124]	34.0 ± 63.9	26.7 ± 65.5	-21%	"	"	"
SDQT	ms	[123]	5.34 ± 4.69	5.14 ± 4.94	-4%	4.52 ± 1.70	6.53 ± 4.31	+44%
QTc_(Bazett)_	ms	[123]	390 ± 30	370 ± 40	-5%	394 ± 26	404 ± 28	+3%
QTVN	-	[123]	0.19 ± 0.14	0.21 ± 0.21	+11%	0.17 ± 0.17	0.51 ± 1.15	+200%
QTVi	-	[123]	-1.35 ± 0.31	-1.34 ± 0.49	+1%	-1.50 ± 0.32	-1.24 ± 0.38	+17%
mBP	mmHg	[24]	82.47 ± 10.35	91.6 ± 12.34	+11%	83.8 ± 11.9	93.9 ± 12.5	+12%
CO	l/min	[24]	6.59 ± 1.2	7.07 ± 1.51	+7%	5.34 ± 0.83	5.71 ± 0.90	+7%
SCL	μS	[25]	5.8 ± 4.3	9.3 ± 5.6†	+60%	11.68 ± 5.25	14.91 ± 4.89	+28%
NSCRpm	1/min	[25]	1.8 ± 2.4	6.9 ± 3.4†	+283%	5.0 ± 2.3	7.1 ± 0.8	+42%
BR	rpm	[124]	15^§^	21^§^	+40%	15.6 ± 4.0	20.4 ± 3.5	+31%

^*^ These results were reported as heart rate HR in bpm and had to be transformed to match RRmean in ms. The non-linearity of the transformation RRmean [ms] = 60 000/HR [bpm] skews the normal distribution. To reduce the conversion error of the standard deviations, they were averaged from the transformed values of the two points ± one standard deviation away from the mean.

^†^ These results were calculated by selecting the maximum value for each participant instead of calculating the mean. As this procedure was only applied to evaluate the MMST phase, changes with regard to baseline may appear larger than in studies that always used the mean.

^§^ These results were only reported graphically. The median value was read from the figure; mean or standard deviation cannot be derived.

Self-assessment showed that the induction of acute mental stress successfully affected SAL and all emotional dimensions of the SAM: valence, arousal, and dominance. Our results for the SAL response were slightly lower but generally in line with values reported in the literature (see Table 6). As the SAM has never before been used in studies applying the MMST, a direct comparison cannot be provided. However, it is apparent that acute mental strain lowers valence and increases arousal. Reduced dominance can be attributed to the uncontrollability included in the stress test.

Before this section proceeds to address RQ1 and RQ2, it should be noted that multiple testing and rather conservative sphericity corrections set the bar for statistical significance high. The low PSQ scores allow for the proposition that mid- to long-term psychosomatic strain did not interfere the results.

Heart rate variability decreased during the acute mental stress phase considering RMSSD (-19%), SDSD (-19%), pNN50 (-28%), NN50 (-21%), and SD1 (-19%). Heart rate variability data reported in the literature fit our observations (see Table 6) with a tendency towards reduced high-frequent fluctuations and a sympathovagal imbalance in favor of sympathetic excitation. In general, however, these changes were not prominent enough to achieve statistical significance after adjustment of significance levels. Furthermore, frequency domain heart rate variability measures reflect them only to a limited extend. The only significant heart rate variability parameter, VLF, largely increased (+27%) while both relative and absolute LF and HF power as well as the LFHFratio remained mostly unaffected (LF: +2%, HF: -1%, LFHFratio: +1%, LFn: +2%, HFn: -2%). While HF is linked to respiratory sinus arrhythmia and therefore to the parasympathetic system, LF is influenced by the cardiac sympathetic and parasympathetic systems as well as, in a subordinate role, by baroreflex activity [115]. Origin and interpretability of the VLF rhythm have been controversial [87]. A more recent review inferred that the VLF rhythm originates from the heart itself and that sympathetic activity modulates its amplitude and frequency [115]. It has been reported that changes in VLF translate into long-term fluctuations of the detrended fluctuation analysis [125], which our observations confirm (DFA2: +30%). We followed the recommendations in [126] and controlled for respiration: BR mostly falls within the HF band (9 to 24 rpm, see Fig 7), which means respiratory sinus arrhythmia mainly modulates frequency domain heart rate variability measures located in this spectral region. Mean BRV changed from 1.4 rpm (0.023 Hz) during the baseline rest phase to 2.4 rpm (0.04 Hz) during the acute mental stress phase. This variability may modulate respiratory sinus arrhythmia but falls within the lower frequency bands. To conclude, several vital signs indicated reduced heart rate variability and reports from the literature fit with our findings. However, not all parameters reflect this, as can be seen in the frequency domain heart rate variability measures, for example.

In contrast to [123], we found acute mental strain to increase QT variability in terms of SDQT (+44%) and QTVN (+200%), reflecting higher sympathetic activation. QTVi is known to increase in response to not only mental but also physical stressors and is treated as an indicator for sympathetic excitation [88]. Our results confirm previous reports from [88] and show an increase of QTVi by +17%. Differences to [123] may occur due to the precision of QT interval extraction methods and sample sizes [88, 109]. Since QTVi reflects both heart rate variability and QT variability, and thus sympathovagal imbalance, we investigated the influence of the RR interval on the QT interval in more detail. Both the RR interval and the QT interval shorten due to acute mental strain. Correction of the heart rate dependence of the QT interval changes this behavior, and QTc shows an increase during the acute mental stress phase. These findings might lead to a better understanding of the independent contribution of sympathetic and parasympathetic tone to the QT interval duration in further studies. To investigate the behavior of ventricular excitation in relation to chronotropic and inotropic changes, we correlated RRmean, QTmean, and QTc to SV (numerical values for QTc_(Bazett)_, but QTc_(Fridericia)_ showed similar behavior). While RRmean (*ρ* = 0.52, *p* < 0.001) and QTmean (*ρ* = 0.42, *p* < 0.001) correlated with SV, QTc did not (*ρ* = 0.07, *p* = 0.53). This means that the relative duration of ventricular excitation remains constant for different levels of heart rate and stroke volume. In summary, it can be stated, that acute mental stress elicited a more pronounced response in QT variability measures than in heart rate variability measures.

The main changes observed in the cardiovascular system were peripheral vasoconstriction, elevated blood pressure, and positive chronotropy. All of these changes indicate excitation of the sympathetic nervous system. Elevated blood pressure and positive chronotropy have been reported in the literature, which is consistent with our findings (see Table 6). Peripheral vasoconstriction can be inferred from the highly significant measure DC. However, this affected the overall vascular resistance only slightly (see TPR), which indicates vasodilation in more central regions such as the brain, skeletal muscles or the heart. The pronounced increase of blood pressure causes the pulse wave to propagate faster throughout the vascular system and explains reduced pulse arrival times. Heart rate increased (see RRmean) while SV remained mostly unaffected, which in the end led to increased CO. The changes in heart rate and blood pressure also reflect in LVWI, which is calculated from CO and mBP [120].

Skin conductance changed markedly due to acute mental strain, which concerns both tonic and phasic activity. Reports of SCL and NSCRpm from the literature are consistent with our results (see Table 6). SCRamp and SCRriseTime have not been previously investigated for the MMST. While the reduction of SCRriseTime due to acute mental strain appears rather subtle, the increase of SCRamp is more substantial. As sweat gland secretion is stimulated exclusively by the sympathetic nervous system [99], the observed skin conductance response can be quite directly attributed to sympathetic excitation.

Respiration altered highly significant in response to acute mental stress. Participants breathed not only much faster (increased BR), but also much more irregularly (increased BRV). Effects of acute mental stress on respiration are mediated via the limbic system (amygdala) and the paralimbic system (anterior cingulate cortex) [94]. While the response of BR to acute mental stress has been extensively studied, with results confirmed by our findings (see Table 6 for example), research addressing the BRV response is still ongoing [94, 127]. Increased variability of the breath rate has been found during mental arithmetic tasks before while sustained attention without stressful stimulation reduced respiratory variability [128].

The regression analysis identified the most important biosignal vital signs (RQ3), the selection of which seems plausible. The selected vital signs belong to different groups (see Table 4), of which only heart rate variability is not represented, and target different organs with a wide range of physiological functions. Heart rate variability is frequently attributed major importance for strain detection, which is not without controversy [126]. Our results relativize the prominent position of heart rate variability measures and point out various effective alternatives directed towards ventricular repolarization, chronotropy, blood pressure, skin conductance, and respiration. Though the results seem plausible, we would like to point out that the logistic regression has not accounted for dependency of observations and possible correlation among vital signs.

The response of salivary cortisol concentration (RQ4) indicated a pronounced activation of the HPA axis, which suggests successful activation of the amygdala. The observed cortisol response matches with prior reports regarding the MMST (peak time: 20 min after stressor onset, effect size Cohen’s *d*: 0.6) [25]. Furthermore, peak time and maximum effect size of the cortisol response both fit the values reported in the meta-analysis by Dickerson and Kemeny [20].

The response of salivary α-amylase concentration (RQ4) exhibited large effect sizes. While the deflection appears clear, the direction, a concentration decrease after stressful stimulation, surprises. However, the relationship between autonomic stimulation and salivary α-amylase concentration is more complex than often portrayed [129] and we did not control for confounding effects such as salivary flow rate, which raises due to parasympathetic stimulation [130]. As α-amylase has never before been included in studies applying the MMST, a direct comparison cannot be provided.

Following the original publications [24, 25], the MMST was analyzed as a single phase and not by its different response times. An influence of the response time on the vital signs is possible and different intensities of hand movement to operate the mouse could induce movement artifacts to varying extends. Also, participants’ experience with meditation routines, deep breathing, mindfulness exercises, and immersive video games was not taken into account, factors that could influence the individual stress-strain relationship [22, 131, 132]. Finally, it should be noted that the participants may have experienced the measurement setup as rather unusual. This might act as an additional stressor, although skin conductance, heart rate, and respiration rate generally indicated successful acclimatization prior to the baseline phase.

In summary, methods from all three domains of strain assessment (see) indicated a pronounced stress response. Acute mental stress caused activation of multiple organ systems. Biosignal vital signs with the clearest responses addressed ventricular repolarization variability, (cardio-)vascular haemodynamics, skin conductance, and respiration. With the exemption of QT variability, our findings are consistent with previously reported results from other works applying the same stress test. In the case of QT variability, we were able to determine the expected changes due to increased sympathetic tone during acute mental stress for the first time with regard to the MMST. Our findings support the hypothesis of the activation of both the sympathetic nervous system and the HPA axis in response to acute mental stress. However, not all vital signs showed such incisive changes, as for example frequency domain measures of heart rate variability.

To the best of the authors’ knowledge, this study was the first to include such a variety of vital signs to assess the human response to acute mental stress. The multimodal dataset of our study offers much potential for further analysis. For example, videos may be used for non-contact measures, e.g. by facial emotion recognition [133] or imaging photoplethysmography [134]. Biosignals targeting different organ systems may be utilized to study effects of acute mental strain on organ interaction with network-physiological approaches [135]. Future research might extend this work by investigation of different types of stressors and factors that could influence the individual stress-strain relationship.

## 6. Conclusions

We provide a fundamental overview over the various methods for controlled induction of stress and non-invasive assessment of strain as well as key aspects of functional physiology on this background. Furthermore, we present a comprehensive multimodal study resulting in the Dresden Multimodal Biosignal Dataset for the Mannheim Multicomponent Stress Test. We found pronounced stress responses across a wide range of different strain assessment methods and identified the most important vital signs measured with biosignals. This work facilitates not only a broader understanding of the assessment of acute mental strain but also provides orientation for further multimodal investigations and practical applications regarding acute mental stress, and thus fosters a more unified assessment of acute mental strain. Our dataset allows for further research in many directions, for example in the fields of facial emotion recognition, imaging photoplethysmography, or network physiological interaction analysis.

## Supporting information

S1 Table Descriptive statistics for the vital signs from biosignals across all phases and RMANOVA test results. Mean ± standard deviation given for each phase. To protect the RMANOVA from outlier distortion, vital signs were filtered with the MATLAB function rmoutliers in its standard configuration. Greenhousse-Geisser correction factor ε_GG_ given if Mauchly’s test indicated violation of the sphericity assumption. Significance markers according to Bonferroni corrected significance levels (*: *p* < 0.05/60, **: *p* < 0.01/60, ***: *p* < 0.001/60). n: Available participants.
(DOCX)

S1 Fig Boxplots for the baseline rest phase and the acute mental stress phase for vital signs without significant t-test results that showed no trend. These are the vital signs not previously included in Figs 7 or 8. *p*-value from two-sample t-test. ×: Mean value.
(TIF)

S1 Appendix Statistics of the binary logistic regression. (DOCX)

S1 Video Excerpt of the MMST test screen. The excerpt contains the change in response times for the arithmetic task (3 s at the beginning, 2 s at the end). Explosive sounds occur with each missing or erroneous answer. Correct answers increase the counter “Stand” by one.
(MP4)
