# Supplementary material for: Assessment of the human response to acute mental stress–An overview and a multimodal study
Source: PLoS One. 2023 Nov 9;18(11):e0294069. doi: 10.1371/journal.pone.0294069 (PMC10635557; doi:10.1371/journal.pone.0294069)
Supplement: S1 Table — Mean ± standard deviation given for each phase. To protect the RMANOVA from outlier distortion, vital signs were filtered with the MATLAB function rmoutliers in its standard configuration. Greenhousse-Geisser correction factor εGG given if Mauchly’s test indicated violation of the sphericity assumption. Significance markers according to Bonferroni corrected significance levels (*: p < 0.05/60, **: p < 0.01/60, ***: p < 0.001/60). n: Available participants. (DOCX) [file pone.0294069.s001.docx]

| **Vital sign** | **Phase 1** | **Phase 2** | **Phase 3** | **Phase 4** | **Phase 5** | **Phase 6** | **n** | **F statistic** | **ε_GG_** | ***p*-value** | |
| --- | --- | --- | --- | --- | --- | --- | --- | --- | --- | --- | --- |
| **Heart rate variability measures** | | | | | | | | | | |  |
| Time domain | | | | | | | | | | |  |
| RRmean [ms] | 885 ± 138 | 811 ± 129 | 873 ± 128 | 869 ± 123 | 868 ± 122 | 872 ± 120 | 49 | F(5,240) = 34.672 | 0.59 | 9.1e-17 *** | |
| SDRR [ms] | 61.1 ± 23.7 | 55.8 ± 17.9 | 61.1 ± 20.3 | 61.7 ± 20.0 | 63.3 ± 20.0 | 62.4 ± 19.0 | 44 | F(5,215) = 2.140 | 0.78 | 0.080 | |
| RRVN | 5.9 ± 5.6 | 5.3 ± 3.3 | 5.6 ± 4.2 | 5.7 ± 4.4 | 6.0 ± 4.2 | 5.7 ± 4.5 | 41 | F(5,200) = 2.312 | 0.75 | 0.065 | |
| STVRR [ms] | 7965 ± 2599 | 7273 ± 2529 | 7586 ± 2689 | 7529 ± 2446 | 7316 ± 2252 | 7222 ± 2377 | 48 | F(5,235) = 4.792 | 0.6 | 0.003 | |
| RMSSD [ms] | 46.0 ± 18.2 | 37.3 ± 16.1 | 43.7 ± 19.9 | 42.5 ± 18.0 | 42.0 ± 17.0 | 41.4 ± 16.8 | 46 | F(5,225) = 9.295 | 0.58 | 1.6e-05 *** | |
| SDSD [ms] | 46.0 ± 18.2 | 37.3 ± 16.1 | 43.7 ± 19.9 | 42.5 ± 18.0 | 42.0 ± 17.0 | 41.4 ± 16.8 | 46 | F(5,225) = 9.296 | 0.58 | 1.6e-05 *** | |
| pNN50 | 0.25 ± 0.17 | 0.18 ± 0.16 | 0.23 ± 0.18 | 0.21 ± 0.17 | 0.20 ± 0.16 | 0.21 ± 0.17 | 44 | F(5,215) = 10.276 | 0.57 | 6.6e-06 *** | |
| NN50 | 75.0 ± 42.7 | 59.1 ± 45.3 | 68.4 ± 45.3 | 64.3 ± 41.8 | 62.0 ± 39.5 | 62.0 ± 41.7 | 50 | F(5,245) = 6.176 | 0.6 | 5.8e-04 * | |
| TRI | 13.76 ± 4.66 | 13.14 ± 3.94 | 13.75 ± 4.20 | 14.34 ± 4.26 | 14.07 ± 3.78 | 14.06 ± 4.32 | 48 | F(5,235) = 2.195 | N/A | 0.056 | |
| TINN [ms] | 218 ± 87 | 205 ± 70 | 219 ± 81 | 225 ± 80 | 223 ± 73 | 218 ± 78 | 45 | F(5,220) = 0.926 | N/A | 0.465 | |
| Frequency domain | | | | | | | | | | |  |
| VLF [s^2^] | 3.59 ± 1.24 | 4.56 ± 0.86 | 3.82 ± 1.26 | 3.99 ± 1.22 | 4.15 ± 1.15 | 4.00 ± 1.18 | 46 | F(5,225) = 8.903 | 0.84 | 7.9e-07 *** | |
| LF [s^2^] | 6.71 ± 1.99 | 6.87 ± 1.72 | 7.18 ± 2.26 | 7.36 ± 2.35 | 7.20 ± 2.00 | 7.19 ± 2.24 | 50 | F(5,245) = 1.574 | 0.67 | 0.193 | |
| HF [s^2^] | 7.30 ± 2.26 | 7.23 ± 2.21 | 6.79 ± 2.11 | 6.64 ± 1.79 | 6.33 ± 1.76 | 6.15 ± 1.71 | 48 | F(5,235) = 6.285 | 0.76 | 1.2e-04 ** | |
| LFHFratio | 1.02 ± 0.50 | 1.03 ± 0.42 | 1.14 ± 0.50 | 1.18 ± 0.51 | 1.22 ± 0.49 | 1.24 ± 0.48 | 44 | F(5,215) = 13.105 | 0.73 | 9.7e-09 *** | |
| LFn | 48.1 ± 10.7 | 49.1 ± 8.5 | 51.0 ± 10.5 | 51.8 ± 10.6 | 53.0 ± 9.5 | 53.3 ± 9.7 | 49 | F(5,240) = 10.911 | 0.74 | 1.5e-07 *** | |
| HFn | 51.9 ± 10.7 | 50.9 ± 8.5 | 49.0 ± 10.5 | 48.2 ± 10.6 | 47.0 ± 9.5 | 46.7 ± 9.7 | 49 | F(5,240) = 10.911 | 0.74 | 1.5e-07 *** | |
| Non-linear | | | | | | | | | | |  |
| ApEn | 1.05 ± 0.13 | 1.12 ± 0.13 | 1.05 ± 0.11 | 1.05 ± 0.14 | 1.04 ± 0.13 | 1.04 ± 0.12 | 46 | F(5,225) = 13.179 | 0.74 | 7.6e-09 *** | |
| DFA1 | 0.73 ± 0.21 | 0.83 ± 0.20 | 0.79 ± 0.23 | 0.79 ± 0.20 | 0.82 ± 0.18 | 0.84 ± 0.24 | 46 | F(5,225) = 2.939 | 0.74 | 0.025 | |
| DFA2 | 0.67 ± 0.33 | 0.87 ± 0.35 | 0.61 ± 0.30 | 0.69 ± 0.40 | 0.71 ± 0.30 | 0.60 ± 0.30 | 47 | F(5,230) = 6.002 | N/A | 3.1e-05 ** | |
| SD1 [ms] | 32.4 ± 12.8 | 26.3 ± 11.4 | 30.8 ± 14.0 | 30.0 ± 12.7 | 29.6 ± 12.0 | 29.2 ± 11.9 | 46 | F(5,225) = 9.289 | 0.58 | 1.6e-05 *** | |
| SD2 [ms] | 79.2 ± 32.6 | 73.7 ± 24.1 | 80.0 ± 26.7 | 81.2 ± 26.9 | 83.9 ± 26.7 | 82.7 ± 25.5 | 46 | F(5,225) = 2.498 | 0.81 | 0.044 | |
| SD1SD2ratio | 0.43 ± 0.16 | 0.37 ± 0.12 | 0.39 ± 0.13 | 0.37 ± 0.12 | 0.36 ± 0.10 | 0.36 ± 0.11 | 45 | F(5,220) = 8.123 | 0.77 | 7.3e-06 *** | |
| **QT variability measures** | | | | | | | | | | |  |
| QT interval and T wave | | | | | | | | | | |  |
| QTmean [ms] | 369 ± 40 | 363 ± 40 | 370 ± 39 | 370 ± 39 | 370 ± 38 | 371 ± 39 | 49 | F(5,240) = 18.249 | 0.6 | 4.1e-10 *** | |
| QTc_(Bazett)_ [ms] | 394 ± 26 | 404 ± 28 | 397 ± 27 | 397 ± 27 | 398 ± 28 | 398 ± 27 | 45 | F(5,220) = 23.702 | 0.47 | 4.5e-10 *** | |
| QTc_(Fridericia)_ [ms] | 385 ± 29 | 390 ± 29 | 388 ± 29 | 388 ± 29 | 388 ± 29 | 389 ± 30 | 50 | F(5,245) = 11.651 | 0.43 | 1.6e-05 *** | |
| Tamp [μV] | 42.3 ± 14.4 | 38.8 ± 13.9 | 42.5 ± 14.9 | 42.3 ± 15.4 | 42.2 ± 15.3 | 42.3 ± 15.1 | 49 | F(5,240) = 8.325 | 0.49 | 1.4e-04 ** | |
| QT variability | | | | | | | | | | |  |
| SDQT [ms] | 4.52 ± 1.70 | 6.53 ± 4.31 | 4.49 ± 1.14 | 4.76 ± 1.47 | 4.92 ± 1.59 | 5.14 ± 2.61 | 39 | F(5,190) = 7.069 | 0.56 | 3.1e-04 * | |
| cSDQT | 2.15 ± 0.65 | 2.98 ± 1.72 | 2.17 ± 0.50 | 2.28 ± 0.65 | 2.37 ± 0.76 | 2.46 ± 1.13 | 40 | F(5,195) = 5.297 | 0.64 | 0.001 | |
| QTVN | 0.17 ± 0.17 | 0.51 ± 1.15 | 0.16 ± 0.07 | 0.18 ± 0.12 | 0.19 ± 0.14 | 0.26 ± 0.58 | 37 | F(5,180) = 9.987 | 0.46 | 5.7e-05 ** | |
| STVQT [ms] | 812 ± 281 | 1188 ± 545 | 775 ± 244 | 820 ± 311 | 834 ± 308 | 858 ± 522 | 43 | F(5,210) = 32.658 | 0.35 | 3.5e-10 *** | |
| LTVQT [ms] | 1362 ± 556 | 2335 ± 1773 | 1421 ± 439 | 1590 ± 685 | 1597 ± 590 | 1631 ± 771 | 40 | F(5,195) = 9.761 | 0.65 | 4.3e-06 *** | |
| QT variability normalized to heart rate variability | | | | | | | | | | |  |
| QTVi | -1.50 ± 0.32 | -1.24 ± 0.38 | -1.51 ± 0.26 | -1.48 ± 0.26 | -1.48 ± 0.25 | -1.46 ± 0.32 | 45 | F(5,220) = 12.413 | 0.61 | 2.6e-07 *** | |
| cQTVi | -2.13 ± 0.30 | -1.90 ± 0.34 | -2.14 ± 0.26 | -2.11 ± 0.24 | -2.11 ± 0.26 | -2.09 ± 0.30 | 44 | F(5,215) = 9.529 | 0.65 | 5.2e-06 *** | |
| QTRRslope | 0.022 ± 0.015 | 0.033 ± 0.034 | 0.023 ± 0.016 | 0.028 ± 0.019 | 0.029 ± 0.018 | 0.028 ± 0.014 | 42 | F(5,205) = 6.939 | N/A | 5.2e-06 ** | |
| VR | 0.12 ± 0.06 | 0.19 ± 0.12 | 0.12 ± 0.09 | 0.12 ± 0.08 | 0.13 ± 0.07 | 0.13 ± 0.09 | 38 | F(5,185) = 21.699 | 0.36 | 1.3e-07 *** | |
| **Haemodynamic measures** | | | | | | | | | | |  |
| SV [ml] | 76.7 ± 14.8 | 75.0 ± 14.5 | 75.3 ± 14.3 | 74.6 ± 14.1 | 74.4 ± 14.7 | 74.4 ± 13.6 | 54 | F(5,265) = 3.526 | 0.46 | 0.026 | |
| SI [ml/m^2^] | 42.1 ± 8.3 | 41.1 ± 8.1 | 41.3 ± 8.2 | 40.9 ± 8.0 | 40.8 ± 8.2 | 40.8 ± 7.6 | 54 | F(5,265) = 3.306 | 0.46 | 0.033 | |
| CO [l/min] | 5.34 ± 0.83 | 5.71 ± 0.90 | 5.24 ± 0.82 | 5.21 ± 0.81 | 5.20 ± 0.85 | 5.22 ± 0.83 | 52 | F(5,255) = 28.461 | 0.35 | 1.4e-09 *** | |
| CI [l/(min*m^2^)] | 2.93 ± 0.46 | 3.13 ± 0.51 | 2.88 ± 0.46 | 2.86 ± 0.45 | 2.85 ± 0.47 | 2.86 ± 0.45 | 52 | F(5,255) = 26.276 | 0.38 | 1.2e-09 *** | |
| TPR [dyn*s/cm^5^] | 1228 ± 241 | 1292 ± 224 | 1356 ± 280 | 1339 ± 266 | 1331 ± 315 | 1305 ± 278 | 53 | F(5,260) = 8.963 | 0.69 | 5.1e-06 *** | |
| TPRI [dyn*s*m^2^/cm^5^] | 2267 ± 593 | 2379 ± 541 | 2499 ± 667 | 2460 ± 602 | 2457 ± 772 | 2399 ± 640 | 46 | F(5,225) = 6.358 | 0.82 | 6.8e-05 ** | |
| dBP [mmHg] | 68.2 ± 11.0 | 76.7 ± 11.5 | 73.4 ± 10.0 | 72.9 ± 8.5 | 72.0 ± 8.6 | 70.4 ± 8.6 | 52 | F(5,255) = 15.773 | 0.8 | 2.8e-11 *** | |
| mBP [mmHg] | 83.8 ± 11.9 | 93.9 ± 12.5 | 89.9 ± 10.5 | 88.2 ± 9.3 | 87.3 ± 9.2 | 85.8 ± 9.4 | 52 | F(5,255) = 20.845 | 0.79 | 3.1e-14 *** | |
| sBP [mmHg] | 108.0 ± 15.5 | 119.3 ± 15.8 | 114.9 ± 13.0 | 112.2 ± 12.0 | 111.1 ± 11.6 | 109.7 ± 12.3 | 52 | F(5,255) = 17.872 | 0.76 | 4.0e-12 *** | |
| ppBP [mmHg] | 39.5 ± 7.4 | 42.6 ± 7.3 | 41.4 ± 6.5 | 39.2 ± 7.2 | 39.1 ± 6.5 | 39.1 ± 6.9 | 50 | F(5,245) = 8.679 | N/A | 1.4e-07 *** | |
| ACI [100/s^2^] | 74.4 ± 21.5 | 73.0 ± 21.2 | 72.2 ± 21.5 | 71.3 ± 21.0 | 70.7 ± 20.7 | 70.4 ± 20.3 | 54 | F(5,265) = 11.068 | 0.56 | 2.5e-06 *** | |
| EDI [ml/m^2^] | 74.2 ± 14.1 | 71.3 ± 12.9 | 73.3 ± 13.7 | 72.8 ± 13.4 | 72.8 ± 13.8 | 72.8 ± 13.0 | 53 | F(5,260) = 5.695 | 0.47 | 0.003 | |
| IC [1000/s] | 50.3 ± 14.6 | 49.6 ± 14.1 | 49.3 ± 14.7 | 48.5 ± 14.3 | 48.7 ± 14.5 | 48.2 ± 13.6 | 54 | F(5,265) = 2.360 | 0.46 | 0.090 | |
| LVET [ms] | 289 ± 26 | 285 ± 30 | 290 ± 27 | 289 ± 25 | 287 ± 25 | 288 ± 26 | 46 | F(5,225) = 1.161 | 0.43 | 0.320 | |
| LVWI [mmHg*l/(min*m^2^)] | 3.27 ± 0.59 | 3.93 ± 0.94 | 3.41 ± 0.64 | 3.32 ± 0.63 | 3.28 ± 0.53 | 3.21 ± 0.53 | 51 | F(5,250) = 31.134 | 0.5 | 2.1e-13 *** | |
| TFC [1/kΩ] | 30.8 ± 5.6 | 31.2 ± 5.7 | 30.9 ± 5.6 | 30.8 ± 5.6 | 30.6 ± 5.7 | 30.6 ± 5.7 | 50 | F(5,245) = 22.057 | 0.5 | 3.5e-10 *** | |
| PATear [ms] | 142 ± 20 | 121 ± 22 | 140 ± 19 | 141 ± 19 | 140 ± 19 | 139 ± 19 | 40 | F(5,195) = 50.223 | 0.37 | 1.0e-13 *** | |
| PATfinger [ms] | 193 ± 17 | 179 ± 22 | 193 ± 17 | 195 ± 17 | 194 ± 18 | 194 ± 18 | 41 | F(5,200) = 33.810 | 0.45 | 1.3e-12 *** | |
| DC | 1.12 ± 0.73 | 1.42 ± 0.64 | 1.37 ± 0.64 | 1.34 ± 0.64 | 1.32 ± 0.62 | 1.29 ± 0.61 | 43 | F(5,210) = 35.120 | 0.37 | 4.1e-11 *** | |
| **Skin conductance measures** | | | | | | | | | | |  |
| SCL [μS] | 11.68 ± 5.25 | 14.91 ± 4.89 | 14.47 ± 5.61 | 13.88 ± 6.31 | 13.44 ± 5.92 | 13.90 ± 6.21 | 53 | F(5,260) = 18.591 | 0.54 | 1.7e-09 *** | |
| NSCRpm [1/min] | 5.0 ± 2.3 | 7.1 ± 0.8 | 5.5 ± 2.1 | 4.9 ± 2.4 | 4.9 ± 2.2 | 5.3 ± 2.2 | 45 | F(5,220) = 20.857 | 0.71 | 1.0e-12 *** | |
| SCRamp [μS] | 0.46 ± 0.41 | 0.76 ± 0.57 | 0.40 ± 0.32 | 0.42 ± 0.41 | 0.48 ± 0.37 | 0.55 ± 0.40 | 48 | F(5,235) = 22.567 | 0.64 | 1.3e-12 *** | |
| SCRriseTime [s] | 2.31 ± 0.48 | 2.24 ± 0.43 | 2.34 ± 0.50 | 2.34 ± 0.49 | 2.35 ± 0.51 | 2.32 ± 0.44 | 52 | F(5,255) = 6.124 | 0.63 | 4.5e-04 * | |
| **Respiration measures** | | | | | | | | | | |  |
| BR [rpm] | 15.6 ± 4.0 | 20.4 ± 3.5 | 15.8 ± 3.9 | 16.2 ± 3.4 | 16.2 ± 3.7 | 16.8 ± 3.6 | 37 | F(5,180) = 33.906 | 0.44 | 5.8e-12 *** | |
| BRV [rpm] | 1.7 ± 0.9 | 2.4 ± 0.8 | 1.9 ± 1.0 | 2.1 ± 0.9 | 1.8 ± 0.7 | 1.9 ± 0.8 | 41 | F(5,200) = 9.231 | N/A | 6.4e-08 *** | |
