# Supplementary figures and images for: Assessment of the human response to acute mental stress–An overview and a multimodal study
Source: PLoS One. 2023 Nov 9;18(11):e0294069. doi: 10.1371/journal.pone.0294069 (PMC10635557; doi:10.1371/journal.pone.0294069)

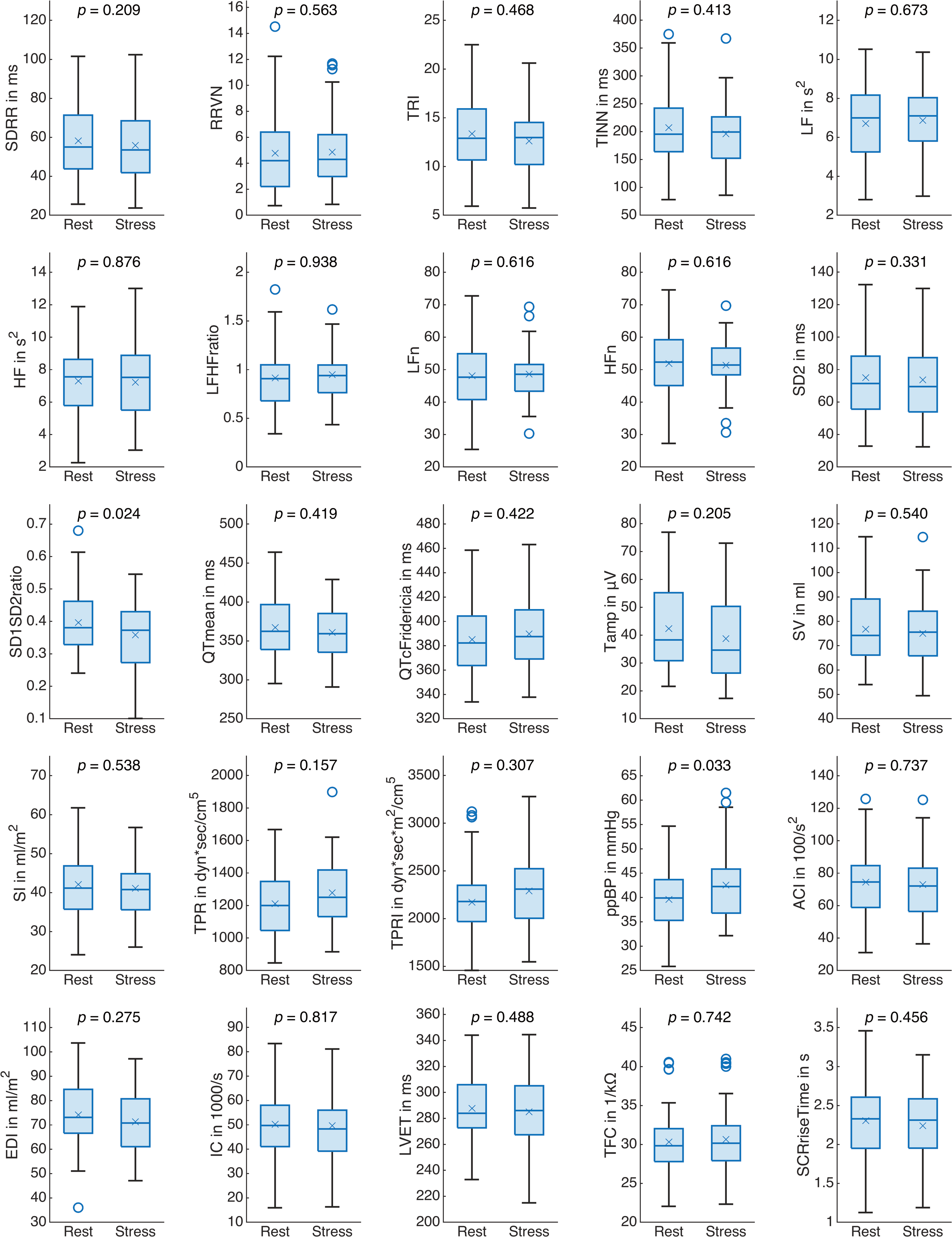

Supplement: S1 Fig — These are the vital signs not previously included in Figs 7 or 8. p-value from two-sample t-test. ×: Mean value. (TIF) [file pone.0294069.s002.tif]
