# Supplementary material for: Assessment of the human response to acute mental stress–An overview and a multimodal study
Source: PLoS One. 2023 Nov 9;18(11):e0294069. doi: 10.1371/journal.pone.0294069 (PMC10635557; doi:10.1371/journal.pone.0294069)
Supplement: S1 Appendix — (DOCX) [file pone.0294069.s003.docx]

This appendix provides statistical results from SPSS 28.0 for the binary logistic regression with the forward selection method (conditional). For each step of the optimization, the omnibus tests of model coefficients, the model summary, the Hosmer and Lemeshow test, the classification table, and the variables in the equation are given.

| **Table A. Omnibus tests of model coefficients.** | | | | |
| --- | --- | --- | --- | --- |
|  | | Chi-square | df | Sig. |
| Step 1 | Step | 141.020 | 1 | 0.000 |
|  | Block | 141.020 | 1 | 0.000 |
|  | Model | 141.020 | 1 | 0.000 |
| Step 2 | Step | 38.213 | 1 | 0.000 |
|  | Block | 179.233 | 2 | 0.000 |
|  | Model | 179.233 | 2 | 0.000 |
| Step 3 | Step | 18.543 | 1 | 0.000 |
|  | Block | 197.776 | 3 | 0.000 |
|  | Model | 197.776 | 3 | 0.000 |
| Step 4 | Step | 23.476 | 1 | 0.000 |
|  | Block | 221.252 | 4 | 0.000 |
|  | Model | 221.252 | 4 | 0.000 |
| Step 5 | Step | 19.159 | 1 | 0.000 |
|  | Block | 240.411 | 5 | 0.000 |
|  | Model | 240.411 | 5 | 0.000 |
| Step 6 | Step | 14.436 | 1 | 0.000 |
|  | Block | 254.847 | 6 | 0.000 |
|  | Model | 254.847 | 6 | 0.000 |
| Step 7^a^ | Step | -1.069 | 1 | 0.301 |
|  | Block | 253.778 | 5 | 0.000 |
|  | Model | 253.778 | 5 | 0.000 |
| Step 8 | Step | 8.241 | 1 | 0.004 |
|  | Block | 262.018 | 6 | 0.000 |
|  | Model | 262.018 | 6 | 0.000 |
| Step 9 | Step | 5.378 | 1 | 0.020 |
|  | Block | 267.397 | 7 | 0.000 |
|  | Model | 267.397 | 7 | 0.000 |
| Step 10 | Step | 4.605 | 1 | 0.032 |
|  | Block | 272.001 | 8 | 0.000 |
|  | Model | 272.001 | 8 | 0.000 |
| ^a^ A negative Chi-squares value indicates that the Chi-squares value has decreased from the previous step. | | | | |

| **Table B. Model summary.** | | | |
| --- | --- | --- | --- |
| Step | -2 Log likelihood | Cox & Snell R Square | Nagelkerke R Square |
| 1 | 204.167^a^ | 0.432 | 0.577 |
| 2 | 165.954^b^ | 0.513 | 0.684 |
| 3 | 147.411^b^ | 0.548 | 0.731 |
| 4 | 123.935^b^ | 0.589 | 0.785 |
| 5 | 104.776^c^ | 0.619 | 0.826 |
| 6 | 90.341^c^ | 0.641 | 0.854 |
| 7 | 91.410^c^ | 0.639 | 0.852 |
| 8 | 83.169^d^ | 0.651 | 0.868 |
| 9 | 77.791^d^ | 0.658 | 0.878 |
| 10 | 73.186^d^ | 0.665 | 0.886 |
| ^a^ Estimation terminated at iteration number 4 because parameter estimates changed by less than .001. | | | |
| ^b^ Estimation terminated at iteration number 6 because parameter estimates changed by less than .001. | | | |
| ^c^ Estimation terminated at iteration number 7 because parameter estimates changed by less than .001. | | | |
| ^d^ Estimation terminated at iteration number 8 because parameter estimates changed by less than .001. | | | |

| **Table C. Hosmer and Lemeshow test.** | | | |
| --- | --- | --- | --- |
| Step | Chi-square | df | Sig. |
| 1 | 19.466 | 8 | 0.013 |
| 2 | 5.686 | 8 | 0.682 |
| 3 | 11.606 | 8 | 0.170 |
| 4 | 3.408 | 8 | 0.906 |
| 5 | 5.437 | 8 | 0.710 |
| 6 | 4.495 | 8 | 0.810 |
| 7 | 6.816 | 8 | 0.557 |
| 8 | 9.368 | 8 | 0.312 |
| 9 | 4.862 | 8 | 0.772 |
| 10 | 6.359 | 8 | 0.607 |

| **Table D. Classification Table.** nState: Binary variable that encodes the condition with 0 for rest and 1 for acute mental stress. | | | | | |
| --- | --- | --- | --- | --- | --- |
| Observed | | | Predicted | | |
|  |  |  | nState | | Percentage Correct |
|  |  |  | 0 | 1 |  |
| Step 1 | nState | 0 | 208 | 0 | 100.0 |
|  |  | 1 | 40 | 1 | 2.4 |
|  | Overall Percentage | |  |  | 83.9 |
| Step 2 | nState | 0 | 202 | 6 | 97.1 |
|  |  | 1 | 23 | 18 | 43.9 |
|  | Overall Percentage | |  |  | 88.4 |
| Step 3 | nState | 0 | 204 | 4 | 98.1 |
|  |  | 1 | 23 | 18 | 43.9 |
|  | Overall Percentage | |  |  | 89.2 |
| Step 4 | nState | 0 | 198 | 10 | 95.2 |
|  |  | 1 | 18 | 23 | 56.1 |
|  | Overall Percentage | |  |  | 88.8 |
| Step 5 | nState | 0 | 198 | 10 | 95.2 |
|  |  | 1 | 17 | 24 | 58.5 |
|  | Overall Percentage | |  |  | 89.2 |
| Step 6 | nState | 0 | 204 | 4 | 98.1 |
|  |  | 1 | 11 | 30 | 73.2 |
|  | Overall Percentage | |  |  | 94.0 |
| Step 7 | nState | 0 | 204 | 4 | 98.1 |
|  |  | 1 | 12 | 29 | 70.7 |
|  | Overall Percentage | |  |  | 93.6 |
| Step 8 | nState | 0 | 204 | 4 | 98.1 |
|  |  | 1 | 11 | 30 | 73.2 |
|  | Overall Percentage | |  |  | 94.0 |
| Step 9 | nState | 0 | 202 | 6 | 97.1 |
|  |  | 1 | 9 | 32 | 78.0 |
|  | Overall Percentage | |  |  | 94.0 |
| Step 10 | nState | 0 | 203 | 5 | 97.6 |
|  |  | 1 | 9 | 32 | 78.0 |
|  | Overall Percentage | |  |  | 94.4 |
| The cut value is .500. | | | | | |

| **Table E. Variables in the equation.** | | | | | | | | | |
| --- | --- | --- | --- | --- | --- | --- | --- | --- | --- |
|  | | B | S.E. | Wald | df | Sig. | Exp(B) | 95% C.I. for EXP(B) | |
|  |  |  |  |  |  |  |  | Lower | Upper |
| Step 1^a^ | QTVi | 1.251 | 0.129 | 93.760 | 1 | 0.000 | 3.493 | 2.712 | 4.499 |
| Step 2^b^ | QTVi | 4.282 | 0.618 | 47.967 | 1 | 0.000 | 72.357 | 21.541 | 243.056 |
|  | LVWI | 1.204 | 0.227 | 28.175 | 1 | 0.000 | 3.335 | 2.138 | 5.203 |
| Step 3^c^ | QTVi | 3.192 | 0.666 | 22.969 | 1 | 0.000 | 24.348 | 6.599 | 89.840 |
|  | LVWI | 1.828 | 0.310 | 34.819 | 1 | 0.000 | 6.220 | 3.389 | 11.413 |
|  | PATear | -0.028 | 0.007 | 15.859 | 1 | 0.000 | 0.972 | 0.959 | 0.986 |
| Step 4^d^ | QTVi | 3.188 | 0.737 | 18.702 | 1 | 0.000 | 24.248 | 5.716 | 102.861 |
|  | LVWI | 1.148 | 0.342 | 11.260 | 1 | 0.001 | 3.152 | 1.612 | 6.162 |
|  | PATear | -0.050 | 0.010 | 27.227 | 1 | 0.000 | 0.952 | 0.934 | 0.969 |
|  | BR | 0.287 | 0.070 | 16.696 | 1 | 0.000 | 1.332 | 1.161 | 1.528 |
| Step 5^e^ | QTVi | 3.261 | 0.811 | 16.156 | 1 | 0.000 | 26.084 | 5.318 | 127.945 |
|  | LVWI | 0.823 | 0.409 | 4.050 | 1 | 0.044 | 2.276 | 1.022 | 5.072 |
|  | PATear | -0.074 | 0.013 | 30.623 | 1 | 0.000 | 0.929 | 0.905 | 0.954 |
|  | NSCRpm | 0.787 | 0.233 | 11.363 | 1 | 0.001 | 2.197 | 1.390 | 3.472 |
|  | BR | 0.254 | 0.081 | 9.858 | 1 | 0.002 | 1.289 | 1.100 | 1.511 |
| Step 6^f^ | QTVi | 3.260 | 0.848 | 14.777 | 1 | 0.000 | 26.047 | 4.942 | 137.276 |
|  | LVWI | 0.483 | 0.465 | 1.079 | 1 | 0.299 | 1.622 | 0.651 | 4.037 |
|  | PATear | -0.073 | 0.015 | 24.353 | 1 | 0.000 | 0.930 | 0.903 | 0.957 |
|  | SCL | -0.259 | 0.076 | 11.541 | 1 | 0.001 | 0.772 | 0.665 | 0.896 |
|  | NSCRpm | 1.318 | 0.295 | 19.973 | 1 | 0.000 | 3.738 | 2.096 | 6.664 |
|  | BR | 0.326 | 0.084 | 15.183 | 1 | 0.000 | 1.386 | 1.176 | 1.633 |
| Step 7^f^ | QTVi | 2.998 | 0.794 | 14.257 | 1 | 0.000 | 20.038 | 4.228 | 94.973 |
|  | PATear | -0.071 | 0.015 | 24.057 | 1 | 0.000 | 0.931 | 0.905 | 0.958 |
|  | SCL | -0.276 | 0.075 | 13.505 | 1 | 0.000 | 0.759 | 0.655 | 0.879 |
|  | NSCRpm | 1.431 | 0.287 | 24.826 | 1 | 0.000 | 4.182 | 2.382 | 7.342 |
|  | BR | 0.363 | 0.079 | 20.987 | 1 | 0.000 | 1.437 | 1.231 | 1.678 |
| Step 8^g^ | QTVi | 2.451 | 0.865 | 8.024 | 1 | 0.005 | 11.599 | 2.128 | 63.229 |
|  | PATear | -0.067 | 0.015 | 19.017 | 1 | 0.000 | 0.935 | 0.907 | 0.964 |
|  | SCL | -0.354 | 0.089 | 15.733 | 1 | 0.000 | 0.702 | 0.590 | 0.836 |
|  | NSCRpm | 1.709 | 0.360 | 22.481 | 1 | 0.000 | 5.522 | 2.725 | 11.190 |
|  | SCRriseTime | -1.886 | 0.713 | 6.988 | 1 | 0.008 | 0.152 | 0.037 | 0.614 |
|  | BR | 0.475 | 0.102 | 21.872 | 1 | 0.000 | 1.609 | 1.318 | 1.964 |
| Step 9^h^ | QTVi | 2.991 | 0.965 | 9.597 | 1 | 0.002 | 19.899 | 3.000 | 131.997 |
|  | LVWI | 1.305 | 0.579 | 5.083 | 1 | 0.024 | 3.687 | 1.186 | 11.466 |
|  | PATear | -0.072 | 0.017 | 18.831 | 1 | 0.000 | 0.931 | 0.901 | 0.961 |
|  | SCL | -0.346 | 0.092 | 14.112 | 1 | 0.000 | 0.707 | 0.590 | 0.847 |
|  | NSCRpm | 1.554 | 0.356 | 19.023 | 1 | 0.000 | 4.728 | 2.352 | 9.503 |
|  | SCRriseTime | -2.600 | 0.822 | 10.013 | 1 | 0.002 | 0.074 | 0.015 | 0.372 |
|  | BR | 0.437 | 0.103 | 17.938 | 1 | 0.000 | 1.548 | 1.265 | 1.895 |
| Step 10^i^ | QTVi | 3.237 | 1.053 | 9.449 | 1 | 0.002 | 25.447 | 3.231 | 200.408 |
|  | LVWI | 1.344 | 0.616 | 4.764 | 1 | 0.029 | 3.834 | 1.147 | 12.816 |
|  | PATear | -0.079 | 0.019 | 18.061 | 1 | 0.000 | 0.924 | 0.891 | 0.958 |
|  | SCL | -0.305 | 0.091 | 11.181 | 1 | 0.001 | 0.737 | 0.616 | 0.881 |
|  | NSCRpm | 1.672 | 0.406 | 16.937 | 1 | 0.000 | 5.321 | 2.400 | 11.796 |
|  | SCRriseTime | -2.985 | 0.918 | 10.564 | 1 | 0.001 | 0.051 | 0.008 | 0.306 |
|  | BR | 0.384 | 0.108 | 12.703 | 1 | 0.000 | 1.469 | 1.189 | 1.814 |
|  | BRV | 0.780 | 0.372 | 4.386 | 1 | 0.036 | 2.181 | 1.051 | 4.525 |
| ^a^ Variable(s) entered on step 1: QTVi. | | | | | | | | | |
| ^b^ Variable(s) entered on step 2: LVWI. | | | | | | | | | |
| ^c^ Variable(s) entered on step 3: PATear. | | | | | | | | | |
| ^d^ Variable(s) entered on step 4: BR. | | | | | | | | | |
| ^e^ Variable(s) entered on step 5: NSCRpm. | | | | | | | | | |
| ^f^ Variable(s) entered on step 6: SCL. | | | | | | | | | |
| ^g^ Variable(s) entered on step 8: SCRriseTime. | | | | | | | | | |
| ^h^ Variable(s) entered on step 9: LVWI. | | | | | | | | | |
| ^i^ Variable(s) entered on step 10: BRV. | | | | | | | | | |
